# Long COVID-19 autoantibodies and their potential effect on fertility

**DOI:** 10.3389/fimmu.2025.1540341

**Published:** 2025-05-27

**Authors:** Laura Talamini, Dennyson Leandro M. Fonseca, Darja Kanduc, Olivier Chaloin, Cindy Verdot, Christian Galmiche, Arad Dotan, Igor Salerno Filgueiras, Maria Orietta Borghi, Pier Luigi Meroni, Natalia Y. Gavrilova, Varvara A. Ryabkova, Leonid P. Churilov, Gilad Halpert, Christian Lensch, Lorenz Thurner, Siew-Wai Fong, Lisa F.P. Ng, Laurent Rénia, Barnaby E. Young, David Chien Lye, José Manuel Lozano, Otávio Cabral-Marques, Yehuda Shoenfeld, Sylviane Muller

**Affiliations:** ^1^ CNRS UMR7242 Biotechnology and Cell Signalling, University of Strasbourg/Strasbourg Drug Discovery and Development Institute (IMS), Strasbourg, France; ^2^ Interunit Postgraduate Program on Bioinformatics, Institute of Mathematics and Statistics (IME), University of São Paulo (USP), São Paulo, Brazil; ^3^ Department of Biosciences Biotechnologies and Biopharmaceutics, University of Bari, Bari, Italy; ^4^ CNRS UPR3572 Immunology, Immunopathology and Therapeutic Chemistry, Institut de Biologie Moléculaire et Cellulaire (IBMC), Strasbourg, France; ^5^ UAR3415, Chronobiotron, Strasbourg, France; ^6^ Zabludowicz Center for Autoimmune Diseases, Sheba Medical Center, Ramat-Gan, Israel; ^7^ Department of Immunology, Institute of Biomedical Sciences, University of São Paulo, São Paulo, Brazil; ^8^ Department of Clinical Sciences and Community Health, University of Milan, Milano, Italy; ^9^ Experimental Laboratory of Immunological and Rheumatologic Researches, Istituto Auxologico Italiano Istituto di Ricovero e Cura a Carattere Scientifico, Milano, Italy; ^10^ Department of Pathology and Laboratory of the Mosaic of Autoimmunity, Saint Petersburg State University, Saint-Petersburg, Russia; ^11^ Pavlov First Saint Petersburg State Medical University, Saint-Petersburg, Russia; ^12^ Research Institute of Phthisiopulmonology, Saint-Petersburg, Russia; ^13^ Department of Pneumology, Allergology and Critical Care Medicine, ECLS Center Saar, Saarland University Hospital, Homburg/Saar, Germany; ^14^ José Carreras Center for Immuno and Gene Therapy and Department of Internal Medicine I, Saarland University, Homburg, Germany; ^15^ ASTAR Infectious Diseases Labs, Agency for Science, Technology and Research (ASTAR), Singapore, Singapore; ^16^ Lee Kong Chian School of Medicine, Nanyang Technological University, Singapore, Singapore; ^17^ School of Biological Sciences, Nanyang Technological University, Singapore, Singapore; ^18^ National Centre for Infectious Diseases, Singapore, Singapore; ^19^ Tan Tock Seng Hospital, Singapore, Singapore; ^20^ Yong Loo Lin School of Medicine, National University of Singapore, Singapore, Singapore; ^21^ Universidad Nacional de Colombia-Sede Bogotá, Departamento de Farmacia, Mimetismo Molecular de los Agentes Infecciosos, Bogotá, DC, Colombia; ^22^ Network of Immunity in Infection, Malignancy, and Autoimmunity (NIIMA), Universal Scientific Education and Research Network (USERN), São Paulo, Brazil; ^23^ Department of Clinical and Toxicological Analyses, School of Pharmaceutical Sciences, University of São Paulo, São Paulo, Brazil; ^24^ Instituto D’Or de Ensino e Pesquisa, São Paulo, Brazil; ^25^ Department of Clinical and Toxicological Analyses, Faculty of Pharmaceutical Sciences, University of São Paulo, São Paulo, Brazil; ^26^ Department of Medicine, Division of Molecular Medicine, Laboratory of Medical Investigation 29, University of São Paulo School of Medicine, São Paulo, Brazil; ^27^ Reichman University, Herzelya, Israel; ^28^ University of Strasbourg Institute for Advanced Study (USIAS), Strasbourg, France

**Keywords:** autoantibodies, peptide sequence identity, male reproductive system, coronavirus infection, post-COVID-19 condition

## Abstract

Impaired spermatogenesis has been reported in coronavirus disease 2019 (COVID-19) patients. However, the impact of severe acute respiratory syndrome coronavirus 2 (SARS-CoV-2) on male fertility remains unclear. The purpose of this multicenter study was to investigate the possible impact of SARS-CoV-2 infection on male fertility and determine the potential reasons leading to impaired male reproductive functions. *In silico* approach identified ~60 amino acid sequences containing at least five continuous residues shared by SARS-CoV-2 Spike glycoprotein and spermatogenesis-linked proteins. Four synthetic peptides were tested with sera from independent cohorts of patients with acute and long COVID-19 syndrome (LCS), and naïve vaccinated subjects. Immunogenicity and pathogenicity studies were performed by immunizing mice with two selected peptides and testing the antigenicity of induced antibodies. While none of four peptides were recognized by antibodies from vaccinated people, infected patients exhibited high reactivity to peptide 4, and LCS patients, especially women, showed elevated antibody levels against peptide 2. Women with LCS and chronic fatigue syndrome had higher levels of peptide 2–reacting antibodies than those with idiopathic chronic fatigue syndrome. Noteworthy, peptide 2 antibodies showed, in *in vitro* experiment, a specific interaction with mouse testicular tissue antigens. These findings raise the possibility that cross-reactive epitopes between SARS-CoV-2 Spike protein and spermatogenesis-related antigens may affect infected patients’ fertility, suggesting a potential for autoimmune responses with human consequences.

## 1 Introduction

Pathological immune hyperactivation induced by infection is a well-known factor that triggers autoimmunity and possibly autoimmune diseases (ADs) in susceptible individuals (1). This scenario is an essential feature of critical illness in coronavirus disease 2019 (COVID-19). Numerous well-documented studies indicate that COVID-19 patients produce autoantibodies and a proportion of them develop ADs (2–7). While autoantibody production seems to develop early during the acute phase of COVID-19, autoimmune manifestations can occur weeks or months later when the symptoms of infection have disappeared and the patient has recovered.

Pre-existing comorbidities, lifestyle, host immune response, genetic background, hormone milieu, and gender contribute to COVID-19 severity, morbidity, and mortality (8, 9). However, in general, patients with autoimmune/auto-inflammatory rheumatic diseases do not exhibit a higher risk of severe acute respiratory syndrome coronavirus 2 (SARS-CoV-2) infection than expected in the global population (10, 11). COVID-19 does not usually seem to trigger clinical flares in autoimmune patients (12), perhaps because the therapy and immune deficit associated with the autoimmune condition do not affect SARS-CoV-2 aggressiveness.

A broad spectrum of circulating autoantibodies has been described in COVID-19 patients. The recurrent questions regarding these autoantibodies concern their origin and whether they may exert pathological effects (12). For decades, these questions also arose in most ADs and are far from being solved. Immune hyperactivation that mainly results from the dramatic secretion of multiple cytokines in SARS-CoV-2–infected patients can enhance the levels of production of all immunoglobulins (Igs), including autoantibodies. This is accompanied by dysregulation of innate and acquired immune responses, especially lymphopenia, reduction of T regulatory cells, over-activation, and then exhaustion of T cells. In addition to immune hyperstimulation, other factors may play a decisive role. Among them, neutrophil extracellular traps (NETs) may be central in the pathogenesis of COVID-19 by promoting a pro-inflammatory response, such as in systemic lupus erythematosus and other ADs, and a pro-coagulant state leading to multi-organ failure (13). The elevated levels of NETs in COVID-19 patients correlate with disease severity and thrombosis.

Molecular mimicry is the leading mechanism by which infectious agents can induce autoimmunity (14). *In silico* studies have identified numerous linear sequences that are shared between sequences of the SARS-CoV-2 nucleocapsid phosphoprotein, membrane/matrix protein, and Spike surface glycoprotein (Spike protein), and human proteins with the self-tissue targets (5, 6, 15). Such investigations represent a critical step in ensuring and enhancing our global knowledge of the possible harmful effects of the cross-reactive adaptive immune response as a post–COVID-19 consequence.

It is well established that the male reproductive system is vulnerable to viral infections. Although the information is still scarce and needs to be completed and reinforced, several reports have documented impaired spermatogenesis in COVID-19 patients. Impairment of sperm quality was found in patients with a moderate disease compared to mild (16–18). It was also mentioned in a case of an asymptomatic patient who was infected by SARS-CoV-2 (19). Another study showed that 25.5% of COVID-19 recoverees were oligo-crypto-azoospermic, significantly higher than the rate in the general population (1%–3%) (20). The reasons for these defects are an elevated immune response in the testis (inflammatory lymphocytic infiltrates, IgG, and pro-inflammatory cytokines), an autoimmune inflammation of the testicles (orchitis) occurring in some COVID-19 patients, the presence of the virus in the testis and viral mRNA in the semen of infected men that might alter seminal parameters, or an alteration of hormonal factors resulting from the infection. Even if we consider that there is no detrimental effect of COVID-19 infection on testicular sperm production, as claimed by independent groups (21), autoimmune response may alter male fertility, due to antibodies against the virus that cross-react with specific antigens of male reproductive organs. The production of such antibodies in women could also provoke preterm delivery or cases of infertility. In this context, the present study aims to determine if antibodies generated by SARS-CoV-2 infection or vaccination could cross-react with spermatogenesis-related antigens, potentially explaining the fertility issues in COVID-19 patients as seen in the context of autoimmunity (22, 23). Patients with acute disease or long COVID-19 syndrome (LCS) and naïve vaccinated individuals who received either the Oxford/AstraZeneca or the Pfizer/BioNTech vaccines were included in this multi-center investigation. We retrospectively examined the serum reactivity with four synthetic peptides selected from a list of ~60 sequences containing at least five consecutive residues shared by SARS-CoV-2 Spike protein and spermatogenesis-linked proteins.

## 2 Materials and methods

### 2.1 Pentapeptides library

To identify pentapeptides that share sequence identity between Spike protein and spermatogenesis-linked proteins, a library of human proteins (in)directly linked to spermatogenesis was assembled from the UniProtKB database (https://www.uniprot.org) (24) using “spermatogenesis” as a keyword. Additionally, the Spike protein primary sequence (ancestral “Wuhan” virus isolate sequence) was split into pentapeptides offset by one–amino acid (aa) residue (that is, MFVFL, FVFLV, VFLVL, and so forth), and the resulting pentapeptides were analyzed for occurrences within spermatogenesis-related proteins. PIR Peptide Match (research.bioinformatics.udel.edu/peptidematch/index.jsp) and Peptide Search (www.uniprot.org/peptidesearch/) programs were used. Occurrences and the corresponding proteins were annotated.

### 2.2 SARS-CoV-2 Spike protein structure analysis

Information on the Spike protein coded by the viral open reading frame 2 and its three‐dimensional (3D) structure coordinates file were obtained (25). 3D structure coordinate files were downloaded from the Protein Data Bank (PDB) site (https://www.rcsb.org). Protein molecular modeling and personalization were performed on the basis of the PDB file coded 6vxx, which represents the Spike protein closed conformation. Peptides 2, 3, and 4 were highlighted on the Spike protein chain A for their localization in its 3D structure. For peptide 1 located at the Spike protein C-terminus, a predictive 3D structure model was generated from the PepFold remote server as described (26, 27). Therefore, PDB files for all peptide sequences were obtained, fulfilling the Ramachandran plot structure requirements, as well as the geometrical constraint and restraint parameters for a valid protein structure. Hence, personalized molecular modeling for displaying all PDB 3D structure coordinate files was allowed using the VMD 1.9.3 version software (NIH Biomedical Research Center for Macromolecular Modeling and Bioinformatics, University of Illinois) (28).

### 2.3 Cohorts of infected patients, vaccinated subjects, and healthy controls

We included 182 adult individuals in this study. Sera from 163 infected or vaccinated patients from different cohorts were obtained from donors after written consent. The main characteristics of infected patients acquired by retrospective chart review are summarized in **Supplementary Table S1**. The most common effects present in patients with LCS included breathlessness, chronic fatigue, “brain fog” anxiety, and stress. No information in the patient’s records regarding the possible signs of autoimmune infertility in men and women (anti-sperm antibodies in men, and premature ovarian failure and ovarian autoantibodies in women) was available. Due to the retrospective nature of the study, data on the pre-existing immunity to SARS−CoV−2, which is possibly linked to past hCoV strains or other sources of cross-reactive immunity to SARS-CoV-2, were also unavailable. As patients with LCS had been infected with SARS-CoV-2 in late summer to winter of 2020, they had not previously been vaccinated. Our study examined the sera of men and women to determine whether the data could differ as a function of gender (the sex of donors is listed in **Supplementary Table S1**).

Eighty-six healthcare workers vaccinated in January to February 2021 with BNT162b2 (Comirnaty/Pfizer-BioNTech) and 45 with ChAdOx1 (Vaxzevria/Oxford-AstraZeneca) were included. Inclusion/exclusion criteria and demographic characteristics of vaccinated subjects have been reported previously (29). Adverse side effects or any clinical manifestation potentially correlated with vaccination were recorded for all the investigated subjects (29). The follow-up of the vaccinated subjects was carried out for 12 months starting from the first injection of the vaccine.

Control sera (n = 19; **Supplementary Table S1**) were randomly selected from a bank of healthy blood donations collected before the worldwide outbreak of COVID-19 (Etablissement Français du Sang, Strasbourg, France). As anonymous blood donation involves no disclosure of the donor’s identity or personal information, no information was available regarding gender and age distribution of voluntary, non-remunerated donors. All serum samples were stored at −20°C or −80°C until use.

### 2.4 Antisera to Spike peptides, purification of specific antibodies

Peptides were synthesized and purified as described (30). Their homogeneity was checked by analytical high-performance liquid chromatography, and their purity was ≥97%. Peptide identity was verified by liquid chromatography–mass spectrometry. Spike peptides were mixed with maleimide-activated keyhole limpet hemocyanin (Sigma, K0383) and given subcutaneously (first administration) and intramuscularly (booster, after 2 weeks) to healthy, inbred strain, BALB/c female mice (100 µg injection/mouse; six mice/peptide). Peptide solutions were mixed 1:1 with complete Freund’s adjuvant (Sigma, ref. F5881) for the first injection and with incomplete Freund’s adjuvant (Sigma, ref. F5506) for subsequent injections. Mice were bled 7 days after the second injection. A control blood sample was collected from each mouse before the first injection. The blood withdrawal was performed under gas anesthesia (O_2_/isoflurane, 4%–5% induction, and 1%–3% maintenance). At the end of the experiment, mice were euthanized by cervical dislocation. Serum anti-Spike peptide antibodies were purified using protein G magnetic beads (Magne Protein G, Promega, G7472) following the manufacturer’s instructions. Purified antibodies were stored without preservatives at −80°C until use.

### 2.5 Enzyme-linked immunosorbent assay

Standard enzyme-linked immunosorbent assay (ELISA) procedures measured antibody reactivity in human and mouse sera. Spike peptides (2 µM) were directly coated onto polyvinyl plates (Thermo Fisher, ref. 2801; 100 µL/well) and incubated overnight at 37°C. Plates were post-saturated with 0.8% (w/v) bovine serum albumin (BSA; Roche Diagnostics, ref. 10735094001) in phosphate-buffered saline (PBS) containing 0.05% (v/v) Tween (PBS-T) for 1 h at 37°C. After washing plates with PBS-T, human or mouse sera, diluted 1:1,000 and 1:500, respectively, in PBS-T–0.8% BSA, were added to plates and incubated for 2 h at 37°C (100 µL/well). Plates were washed before applying (100 µL/well) goat anti-human or goat anti-mouse IgG Fcγ–horseradish peroxidase (HRP) conjugates (Jackson ImmunoResearch Laboratories, refs. 109-035–008 and 115-035-008, respectively) diluted in PBS-T (1:20,000 and 1:15,000, respectively) to detect antibody binding. After incubation for 1 h, plates were washed twice with PBS-T and twice with distilled water. The final reaction was visualized with H_2_O_2_ used as substrate and 3,3′,5,5′-tetramethyl benzidine (Sigma, ref. T2885-SG) used as the chromogen (75 µL/well). After 30 min at 37°C, the enzymatic reaction was stopped by adding 25 μL of 1 N HCl. Absorbance was measured at 450 nm on a Thermo Scientific Multiskan. Absorbance values were corrected by subtracting the extinction values from the plate material measured at 535 nm. All serum samples were systematically tested in at least two independent assays. Each plate contained several samples from healthy individuals to determine baseline absorption and possible false reaction. The threshold for positivity was determined as the mean absorbance value +2 SD of the control sera.

### 2.6 Western blotting

The full-length recombinant SARS-CoV-2 (2019-nCoV) Spike (S1 + S2) protein containing a histidine (His)–tag at its C-terminal end (BioLegend, ref. 793706) was mixed with Laemmli buffer (Bio-Rad, ref. 1610737) and denatured for 10 min at 95°C. The protein (1–2 µg) and molecular weight marker (Bio-Rad, ref. 1610393) were run on 4%–20% Mini-PROTEAN TGX Gels and transferred onto Trans-Blot Turbo nitrocellulose membrane (Bio-Rad). Rabbit IgG (1 µg) specifically recognized by HRP-conjugated anti-rabbit IgG antibody was included as a negative control. Following the saturation with blocking agent, blots were incubated with the primary antibodies raised in mice or anti-His monoclonal antibody (0.3 µg/mL; Invitrogen, MA1-21315) overnight at 4°C. HRP-conjugated goat anti-mouse IgG (1:15,000; Jackson ImmunoResearch, 115-035-008) or HRP-conjugated goat anti-rabbit IgG (1:15,000; Jackson ImmunoResearch, ref. 111-035-008) secondary antibody and Clarity Western ECL Substrate (Bio-Rad, ref. 1705060) were used to reveal the signal through the ChemiDoc XRS System (Bio-Rad).

### 2.7 Immunofluorescent staining

Testes from a healthy 10-week-old C57BL/6 mouse were collected, fixed in 4% (v/v) paraformaldehyde for 24 h, incubated in 30% (w/v) sucrose overnight, snap-frozen, and stored at −80°C. Cryosections (7 µm) were cut, permeabilized in PBS–0.1% (v/v) Triton X-100 for 5 min, briefly rinsed in PBS, and blocked in PBS–0.1% Triton X-100–3% BSA for 1 h. Sections were incubated with primary antibodies: anti–peptide 2 or mouse IgG control (Sigma, ref. I5381), diluted 1:20 in PBS–Triton X-100–BSA blocking buffer overnight at 4°C. After washing, AF488-labeled goat anti-mouse IgG (Invitrogen, ref. A11029) was applied for 1 h at room temperature, and nuclei were stained with Hoechst 33258 for 10 min (2 µg/mL in PBS; Invitrogen, ref. H3560) before mounting cover slips with Fluoromount Aqueous Mounting Medium (Sigma, ref. F4680). Images were acquired using a Leica DMRA2 microscope.

### 2.8 Statistical and bioinformatic analyses

Statistical tests were performed using GraphPad Prism 6 (GraphPad Software Inc.). The statistical tests applied are described in the corresponding figure legends. The statistical significance was assessed by the Mann–Whitney non-parametric test. All data presented (mean ± SD) were obtained from at least two independent experiments. Heatmap graphs were constructed using the ComplexHeatmap R package (https://www.bioconductor.org/packages/release/bioc/html/ComplexHeatmap.html) and the Morpheus tool (https://software.broadinstitute.org/morpheus). Regression analysis was done using GAMLSS R packages with ZAGA (Zero Adjusted Gamma Distribution; https://search.r-project.org/CRAN/refmans/gamlss.dist/html/ZAGA.html).

## 3 Results

### 3.1 Identification pentapeptides shared between SARS-Cov-2 Spike protein and human spermatogenesis proteins

Initially, we defined a list of pentapeptides common to human proteins linked to spermatogenesis and SARS-CoV-2 Spike protein. A library of 667 human proteins linked to spermatogenesis was assembled randomly from the UniProtKB database using “spermatogenesis” as a keyword (**Supplementary Table S2**). In parallel, the primary sequence of Spike protein was dissected into pentapeptides offset by one-aa residue. The resulting pentapeptides were analyzed for occurrence within spermatogenesis-related proteins using the PIR Peptide Match and Peptide search programs. Peptide matching analyses led to establishing a list of 217 proteins sharing 245 pentapeptides with the Spike protein for 324 pentapeptide occurrences (**Supplementary Table S3**). Given the size of the viral *versus* human pentapeptide overlap, analyses were limited to 20 protein alterations that had been documented to be related to spermatogenesis disorders using Online Mendelian Inheritance in Man and/or PubMed (**Supplementary Table S4**). This list was further sorted using the Immune Epitope DataBase resource (www.iedb.org/), which includes experimentally validated immunoreactive, Spike protein epitopes (**Supplementary Table S5**) (26).

**Figure 1:**
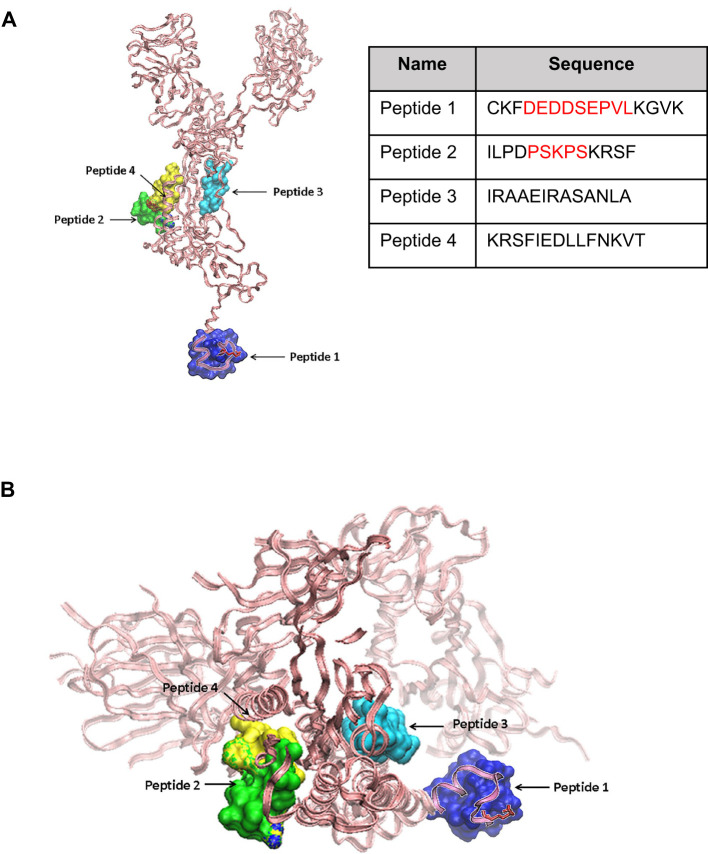
Molecular models of SARS-CoV-2 Spike protein and relative location of peptide sequences targets. **(A)** Side view of the SARS-CoV-2 Spike protein chain A backbone shown in pink pale ribbons from the Protein Data Bank file 6vxx code. Peptides 1 to 4 (the sequence of each is shown) are highlighted in the Spike protein 3D structure. Peptides 2, 3, and 4 (green, cyan, and yellow in solid surface volume representations). A molecular predicted model for peptide 1 from PepFold is shown in a blue solid surface volume and superimposed to the chain A 3D structure for its relative localization. **(B)** A Spike peptide view from the bottom highlighting peptides 1 to 4.

From the 58 segments encompassing contiguous five-mer-long sequences shared between Spike protein and spermatogenesis-related proteins shown in **Supplementary Table S5**, four peptides named 1 to 4 were finally selected for synthesis and testing (**Figure 1**). Peptide 1 (16 aa; Pi = 4.2) encompassed a long sequence of nine residues (DEDDSEPVL) shared between the C-terminus of Spike protein and spermatogenesis-related proteins. Peptide 2 (13 aa; Pi = 10.6) contained a stretch of five shared residues (PSKPS) in the testis-specific serine/threonine-protein kinase 1, essential for male fertility. The sequence of peptide 3 (13 aa; Pi = 10.9) was contiguous to a motif shared between Spike protein and a spermatogenesis-related protein but did not encompass shared residues. Likewise, peptide 4 (14 aa; Pi = 9.9) did not contain a sequence shared between Spike protein and a spermatogenesis-related protein but encompassed the motif IEDLL that is present in histone-lysine N methyltransferase 2D (KMT2D), which is linked to oogenesis (IEDB ID 1309578) (31). Peptides 4 and 2 were overlapping by four residues (KRSF) in the primary sequence of Spike protein. Peptide 2, 3, and 4 are located on the Spike protein chain A. The 3D molecular model of Spike protein and the localization of peptides 1 to 4 are depicted in **Figures 1A, B**.

### 3.2 IgG antibody reactivity to spermatogenesis-related peptides in the serum of COVID-19 patients and vaccinated individuals

**Figure 2:**
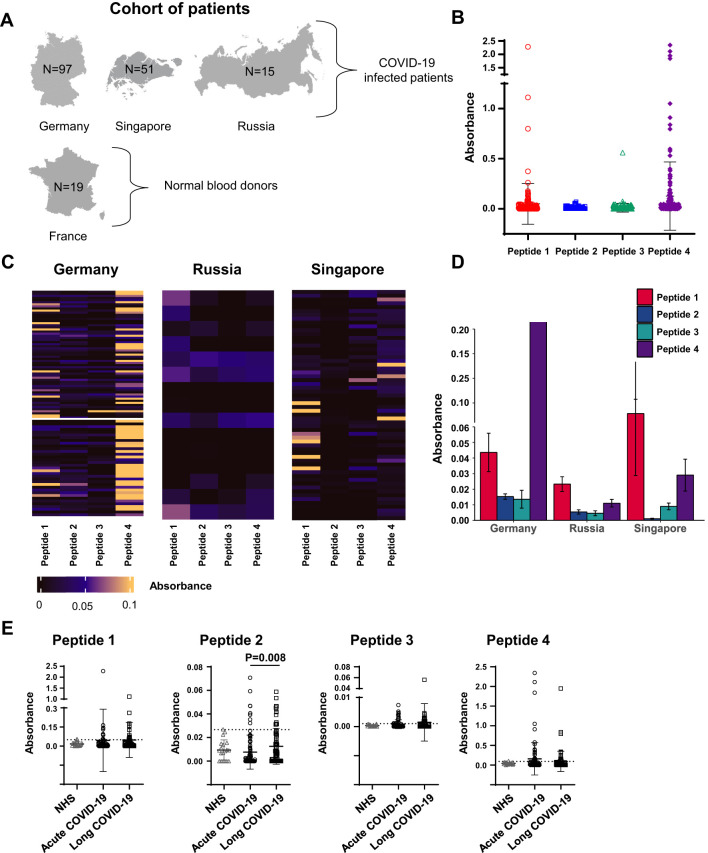
Levels of serum reactivity with peptides 1 to 4 of the SARS-CoV-2 Spike protein categorized by country and disease groups. **(A)** Schematic representation of the study cohort showing the number of individuals by disease group for each country. **(B)** Dot plot showing the levels of serum reactivity to peptides 1 to 4 of all infected individuals. **(C)** Heatmaps showing the levels of serum reactivity to peptides 1 to 4 per country of each patient. The intensity of the reaction is indicated by colors as indicated. **(D)** Bar graphics showing the levels (ELISA absorbance values) of peptide-reacting antibodies by country. **(E)** Dot plot showing the levels of serum reactivity to peptides 1 to 4 distinguishing patients with acute versus long COVID-19. NHS (normal human sera from healthy donors) were used as controls. The dashed line in each bar graphic denotes the cutoff value for each peptide. Data are reported as mean ± SD. Statistical analysis is determined by non-parametric Kruskal-Wallis following Dunn’s test. Differences were considered significant when P < 0.05.

To evaluate the antigenicity of peptides 1–4, we recruited cohorts of adult individuals from different countries, either patients presenting COVID-19 infection proved by nasopharyngeal swabs or polymerase chain reaction test (n = 163) or healthy donors (n = 19) (**Figure 2A**, **Supplementary Table S1**). Infected patients were categorized into two groups corresponding to acute COVID-19 (n = 87) or LCS (n = 76), on the basis of infection severity and length. In these two groups, the mean age was 53 and 42 years, respectively (**Supplementary Tables S1A, B**). The repartition of men and women was found to be different in the two groups, namely, 78% men/22% women in the acute group and 37% men/63% women in the LCS group (**Supplementary Tables S1A, B**). The sera from healthy donors were used to calibrate the test conditions, determine the threshold for positivity [cutoff optical density (OD) values], measure the inter- and intra-assay coefficients of ELISA variability, and verify the absence of unwanted reactivity. Following these calibration steps, the peptide reactivity of sera was accurately evaluated. IgG reactivity was investigated because IgM data are more prone to false positivity, mainly due to test sensitivity and misinterpretation.

As expected, sera of healthy subjects showed low and infrequent (<5% of subjects) reactivity with peptides 1–4, while reactivity with these peptides was found in patients’ sera (**Figure 2**). Patient’s sera reacted with variable levels of intensity (**Figure 2B**); the highest antibody reactivity was observed in the German cohort, mostly with peptide 4, followed by that of sera collected in Singapore, primarily with peptide 1 (**Figures 2C, D**). Although statistically significant, the ELISA absorbance values were generally low with peptide 2, in contrast to absorbance values measured with peptide 4.

Next, the sera reactivity with peptides 1–4 was examined in acute or long COVID-19 patients. Infected patients showed the highest serum reactivity with peptide 4 both in acute and long form (mean OD = 0.15, frequency of 25%; and mean OD = 0.093, frequency of 21%, respectively). Additionally, we found that sera of LCS patients showed significantly higher levels of antibodies against peptide 2 compared to acute COVID-19 individuals’ sera (mean OD = 0.012, frequency of 15.8%, *versus* mean OD = 0.007, frequency 9%, P = 0.008, for acute disease and LCS, respectively; **Figure 2E**).

**Figure 3:**
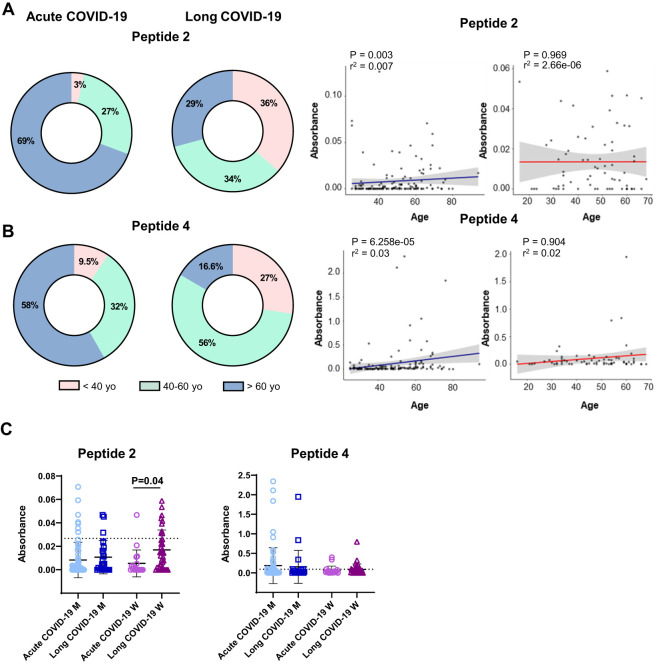
Levels of serum reactivity with peptides 2 and 4 of the SARS-CoV-2 Spike protein according to groups of age, gender, and disease status (acute or long COVID-19 indication). Donut graphs showing the distribution of serum reactivity levels of **(A)** peptide 2 and **(B)** peptide 4 between acute and long COVID-19 patients classified by age (left panels). Scatter plot showing the age distribution of serum levels. Blue lines represent acute COVID-19, and red lines show long COVID-19 group (right panels). The R-squared and adjusted P-values from the Kendall correlation are displayed at the top of the graphs. **(C)** Dot plot reporting serum level of groups described above according to gender. Results are reported as mean ± SD. A dashed line denotes the cutoff value determined for each peptide. Statistical analysis is determined by the Mann–Whitney non-parametric test. Differences were considered significant when P < 0.05. M, men; W, women; yo, year-old.

Based on these results with peptides 2 and 4, we further characterized certain features of COVID-19 patients with and without peptide-reactive antibodies (**Figure 3**, **Supplementary Figures S1**, **S2**). Acute COVID-19 patients with positive antibodies to peptides 2 and 4 were mainly over 60 years old (69% and 58%, respectively), at variance with LCS patients expressing positive antibodies, who were younger (**Figures 3A, B**). Considering the gender of our patients’ cohort and the severity of viral infection, we found that women affected by LCS significantly presented a higher rate of peptide 2 antibodies compared to those with acute forms of COVID-19 (mean OD = 0.017, frequency of 24%, *versus* mean OD = 0.005, frequency 5%, for LCS and acute COVID-19, respectively; P = 0.04). In contrast, we observed only a trend of a higher level of peptide 4 antibodies in men affected by acute COVID-19 compared to LCS (mean OD = 0.18, frequency of 28%, *versus* mean OD = 0.15, frequency of 21%, P = 0.35, in patients with acute disease and LCS, respectively; **Figure 3C**).

The possible association of peptide-reactive antibodies with some pathological characteristics of SARS-CoV-2–infected patients was also analyzed. Detailed information was not systematically listed in the patient’s clinical records to which we had access for our analyses. Based on the available data, no statistically significant correlations emerged concerning to typical biological parameters, e.g., the rate of reactive protein C, levels of total IgG and IgM antibodies, and pre-existing serum autoantibodies to classical autoantigens (e.g., double-stranded DNA, small nuclear ribonucleoproteins, and extractable nuclear antigens Ro/La). No correlation was found with documented clinical features, such as respiratory or neurological impairment (loss of memory), headache, and fever.

**Figure 4:**
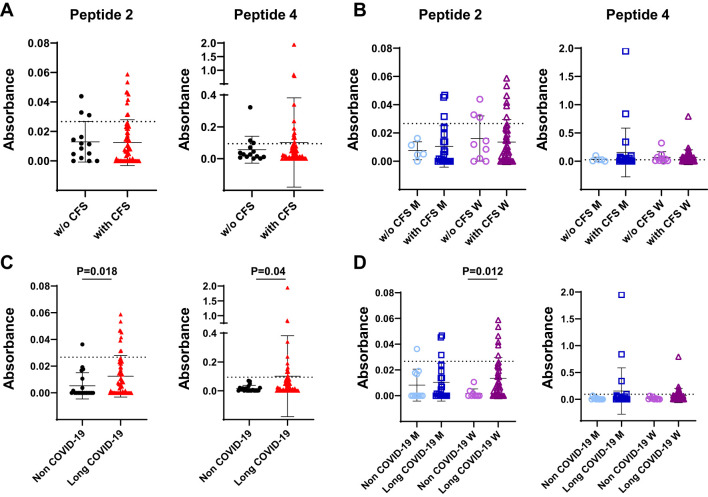
Levels of serum reactivity with peptides 2 and 4 of the SARS-CoV-2 Spike protein according to clinical features of infected patients. **(A)** Dot plot showing serum levels in long COVID-19 patients affected by chronic fatigue syndrome (with CFS) or unaffected (w/o CFS). **(B)** Data in **(A)** according to gender. **(C)** Dot plot showing serum levels in uninfected individuals presenting idiopathic CFS (non–COVID-19 subjects) and in long COVID-19 patients who developed CFS after virus infection. **(D)** Results in **(C)** according to gender. Results are reported as mean ± SD. The cutoff value of each peptide is denoted as a dashed line. Statistical analysis is determined by Mann–Whitney non-parametric test. Differences were considered significant when P < 0.05. M, men; W, women; w/o, without.

No correlation could be established when we considered patients with LCS presenting or not chronic fatigue syndrome (CFS) as a consequence of SARS-CoV-2 infection (**Figure 4A**), regardless of gender (**Figure 4B**). Interestingly, when LCS patients with CFS were compared to COVID-19 negative subjects presenting idiopathic CFS, a significant correlation could be made about the presence of antibodies reacting with peptides 2 and 4 (peptide 2: non COVID-19 mean OD = 0.0053, frequency of 5%, *versus* long COVID-19 mean OD = 0.012, frequency of 14.5%, P = 0.018; peptide 4: non-COVID-19 mean OD = 0.015, frequency of 0%, *versus* long COVID-19 mean OD = 0.10, frequency 21%, P = 0.04) (**Figure 4C**). Furthermore, the sera of women infected by SARS-CoV-2 possessed significantly increased levels of antibodies reacting with peptide 2 compared to women with idiopathic CFS (15% *versus* 0%; P = 0.012; **Figure 4D**).

A similar analysis was also performed using the sera from 131 vaccinated Italian individuals. We tested 90 sera from 45 healthcare workers pre- and post (1 month)–Oxford/AstraZeneca vaccination and 172 sera from 86 healthcare workers pre- and post (3 months)–Pfizer/BioNTech vaccination (**Table 1**). No antibody reactivity was detected in the sera from 131 pre-vaccinees with peptides 1 and 2. Some reactivity with peptides 3 and 4 was measured pre-vaccination in healthcare workers. However, post-vaccination, the percentage of sera reacting with any of the peptides 1–4 was low, and their level of reactivity was extremely weak (**Table 1**). Results lead to conclude that the pattern of sera’s reactivity against Spike protein peptides 1–4 was not found in vaccinees at least 1–3 months post-vaccination with either Oxford/AstraZeneca or Pfizer/BioNTech vaccines.

**Table 1:** Level of serum reactivity with peptides 1 to 4 of the SARS-CoV-2 Spike protein in vaccinated subjects.

Cohort		Peptide 1	Peptide 2	Peptide 3	Peptide 4
Astra Zeneca					
Pre-vaccination (n = 45)	Mean (%)	0.0067 (0%)	0.024 (4.4%)	0.023 (17%)	0.036 (8.8%)
1 month post-vaccination (n = 45)	Mean (%)	0.004 (0%)	0.0054 (4.4%)	0.0046 (17%)	0.0054 (2.2%)
Pfizer					
Pre-vaccination (n = 86)	Mean (%)	0.0071 (1.1%)	0.0045 (1.1%)	0.05 (92%)	0.054 (12.8%)
3 months post-vaccination (n = 86)	Mean (%)	0.0072 (5.8%)	0.0059 (3.5%)	0.0043 (11.6%)	0.0164 (4.6%)

Sera from naïve (healthy) individuals were collected pre- and post-vaccination with adenovirus-based (ChAdOx1, Vaxzevria/Oxford/AstraZeneca) or messenger RNA- (BNT162b2, Comirnaty/Pfizer/BioNTech) vaccines. The table reports average OD values and % positive reactivity with peptides 1 to 4 of the Spike protein in each group. IgG responses only were measured.

### 3.3 *In vitro* characterization of Spike glycoprotein and spermatogenesis-associated antigens cross-reacting antibodies

**Figure 5:**
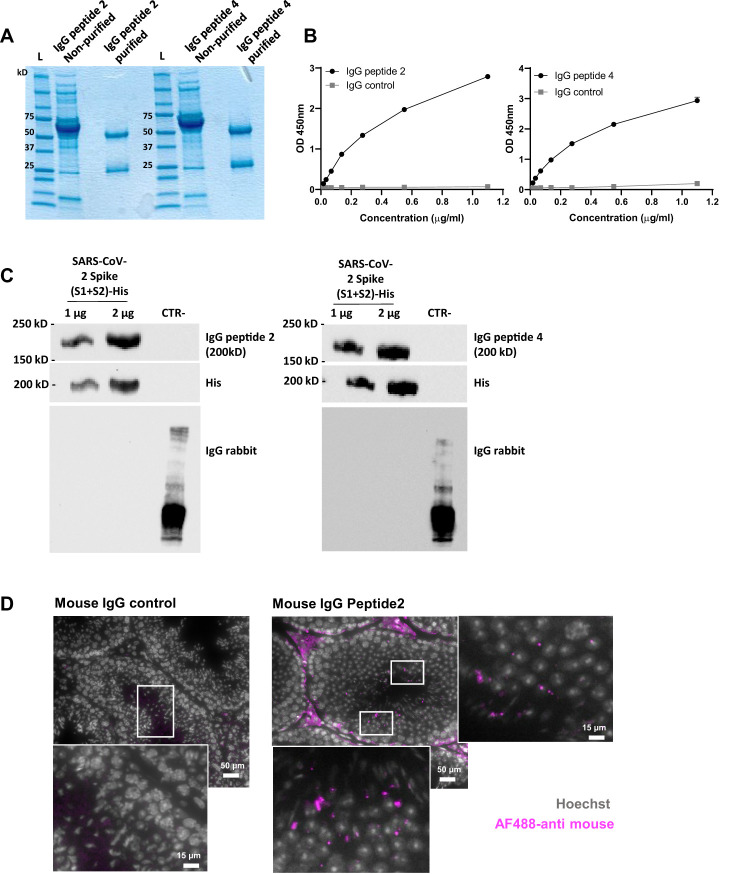
Characterization of IgG antibodies generated in normal mice against peptides 2 and 4. **(A)** Coomassie blue staining gel showing IgG antibodies to peptides 2 and 4 before and after purification on protein G magnetic beads. **(B)** Reactivity in ELISA of IgG antibodies to peptides 2 and 4 towards their homologous (immunogen) peptide. Plates were coated with 2µM of Cys-peptide 2 or Cys-peptide 4 in bicarbonate/carbonate buffer (pH 9.6), and the reactivity of purified IgG antibodies was tested with increasing concentrations (0 to 1.2 µg/mL) of each of them. Normal mouse IgG was used as a control. Data are reported as mean ± SD of absorbance measured at 450 nm. **(C)** Western blots showing the reactivity of purified IgG to peptides 2 and 4 with the His tag-SARS-CoV-2 Spike protein. A control protein (CTR), lacking the His tag and produced in another species, has been added to the gel as a negative control. Membranes were blotted for IgG antibodies at 1 µg/mL, or for anti-6x His tag at 0.7 µg/mL. IgG anti-mouse and anti-rabbit HRP at 0.05 µg/mL were used as secondary antibodies. **(D)** Representative histological images of mouse testicular tissue stained with mouse anti-peptide 2 IgG or normal mouse IgG used as control, and revealed with AF488-labeled goat anti-mouse Ig (magenta). Nuclei were stained with Hoechst (grey). Scale bar = 50 µm or 15 µm (higher magnification).

To characterize the potential functional role of cross-reactive antibodies, we finally immunized healthy mice with peptides 2 and 4. No macroscopic alterations were observed in organs, tissues, or body cavities post-immunization, indicating that neither the peptides nor the induced antibodies exhibited apparent toxicity in BALB/c mice. The generated antibodies were affinity-purified, and their purity was validated through biochemical analyses (**Figures 5A, B**). The specific binding of purified IgG antibodies to peptides 2 and 4 with Spike protein was also confirmed by western blot (**Figure 5C**). The reactivity of IgG antibodies to peptide 2 was further studied by immunochemistry using mouse testicular tissues that we incubated with these antibodies or with normal mouse IgG used as control (**Figure 5D**, **Supplementary File 1**). While no specific signal was observed with control IgG, histological images pointed out a specific one in testicular tissue treated with IgG antibodies to peptide 2, indicating that these antibodies readily react with antigens expressed by mouse testicular tissues.

## 4 Discussion

This study shows that SARS-CoV-2 Spike protein shares several peptide sequences with human spermatogenesis-linked proteins that, if altered, may lead to oligospermia, testicular azoospermia or atrophy, and sterility. Such cases of mimicry are not unique to SARS-CoV-2 and have been described in the case of mumps virus (paramyxoviruses) infection (32). Using four 13- to 16-residue-long synthetic peptides designed from a list of sequences containing at least five identical continuous residues shared by SARS-CoV-2 Spike protein and spermatogenesis-linked proteins, we found that peptides 1 and 2 and, especially, peptide 4, were recognized by IgG antibodies developed in COVID-19 patients. Some differences in intensity and specificity found between cohorts may be due to clinical, genetic and management factors. Peptide 2 showed the most interesting and discriminant results. Patients with LCS, especially women, presented higher levels of IgG antibodies reacting with peptide 2 compared to patients with acute disease. Furthermore, women with LCS and CFS had significantly higher antibody levels than those with idiopathic CFS not caused by an underlying medical condition. A limitation of this study is its retrospective nature, which may introduce selection bias. Additionally, although the COVID-19 sample size was relatively large, the size of certain subgroups was relatively small, which leads us to interpret the results with caution.

In this work, we used pentapeptides as a scanning probe because the literature indicates that a stretch of five aa residues is immunologically endowed with antigenicity and immunogenicity and is a minimal determinant in humoral and cellular immune recognition (33, 34). It is worth noting that the sequence PSKPS in peptide 2 is also present in the testis-specific serine/threonine-protein kinase 1, which associated with HSP90, and is essential in late-phase spermatogenesis and male fertility (35, 36). Peptide 4 contains two remarkable motifs: a KRSF sequence (also present in peptide 2), found in 90 human proteins, including those linked to oogenesis, placenta, and/or decidualization (31), and IEDLL motif contained in an experimentally validated epitope-containing peptide of the SARS CoV-2 Spike protein (3) and present in KMT2D/MLL4, linked to oogenesis (31). MLL4 is notably required for anterior visceral endoderm migration (37). We demonstrated that antibodies to peptides 2 and 4 readily recognize the SARS-CoV-2 Spike protein and mouse testicular antigens (antibodies to peptide 2). However, we could not determine if affinity-purified antibodies to these peptides bind the above-mentioned spermatogenesis- or oogenesis-related proteins, as we were unable to obtain them as pure proteins.

Another study’s central result is that women with COVID-19 develop IgG antibodies to spermatogenesis-related antigens. This discovery could have pathological consequences that we did not suspect from pre-existing literature data. These cross-reactive antibodies, which *de facto* cannot be called “autoantibodies” in women, might damage ovarian function and influence fertility rate years after SARS-CoV-2 infection (38), as anti-Spike IgG antibodies remain detectable one year after hospitalization of COVID-19 patients (39). Men are immunologically tolerant to epitopes of spermatogenesis-related antigens, whereas women could develop more efficiently a memory response. Seminal anti-sperm antibodies, often occurring in autoimmune conditions or post-vasectomy, can cause immunologic infertility by affecting sperm motility, cervical mucus interaction, and other processes (40, 41).

Vaccinated individuals did not develop antibodies reacting with any of the four selected peptides. This difference between infection and vaccination could stem from the absence in vaccinated people of the hyper-inflammatory state and the B-cell compartment hyper-stimulation reported in COVID-19 patients (1). It is also possible that the Spike protein sequences “viewed” by the immune system are not similar in the virus and the antigenic protein resulting from vaccination. Both Pfizer/BioNtech’s messenger RNA-based and Oxford-AstraZeneca’s adenovirus vector vaccines did not induce antibodies to peptides 2 and 4, suggesting that individual component rather than the Spike glycoprotein structure, may affect cross-reactive immune responses. Our samples were collected 1–3 months post-vaccination and not a longer time; therefore, the absence of response to Spike peptides sharing identity with spermatogenesis-related antigens is reassuring in the context of vaccination. Our findings align with data showing that vaccinated healthcare workers do not develop antibodies reacting with cardiolipin, β2 glycoprotein I, phosphatidylserine/prothrombin, and others after vaccination (29).

Antibodies reacting with self-antigens frequently occur in healthy individuals without harmful effect, and most autoantibodies subsets found in autoimmune individuals are devoid of intrinsic pathogenicity. Pathogenic effects of autoantibodies with some manifestations of ADs have been unambiguously demonstrated in a few cases only (42). Here, we showed that affinity-purified antibodies to peptide 2 react with antigens expressed by mouse testicular tissue. The peptide cross-reactivity identified in this study could help predict possible long-term effects of COVID-19 on fertility rate that may be revealed years after SARS-CoV-2 infection. Although much more work should be done to deepen our knowledge, the peptide cross-reactivity, identified in this study, might help predict possible long-term effects of COVID-19 on fertility rate that may be revealed years after SARS-CoV-2 infection. This finding supports the long been recognized hypothesis that cross-reactivity between pathogen-related sequences and self-antigens remains an unquestionable mechanism that triggers autoimmune responses. The fact that million people have been infected by SARS-CoV-2 worldwide inclines us to explore as much as possible the potential secondary, long-term effects that may emerge at a distance from infection itself.

## Data Availability

The original contributions presented in the study are included in the article/**Supplementary Material**. Further inquiries can be directed to the corresponding author.
