# Supplementary material for: Long COVID-19 autoantibodies and their potential effect on fertility
Source: Front Immunol. 2025 May 27;16:1540341. doi: 10.3389/fimmu.2025.1540341 (PMC12149208; doi:10.3389/fimmu.2025.1540341)

# #1 Mouse IgG Peptide 2

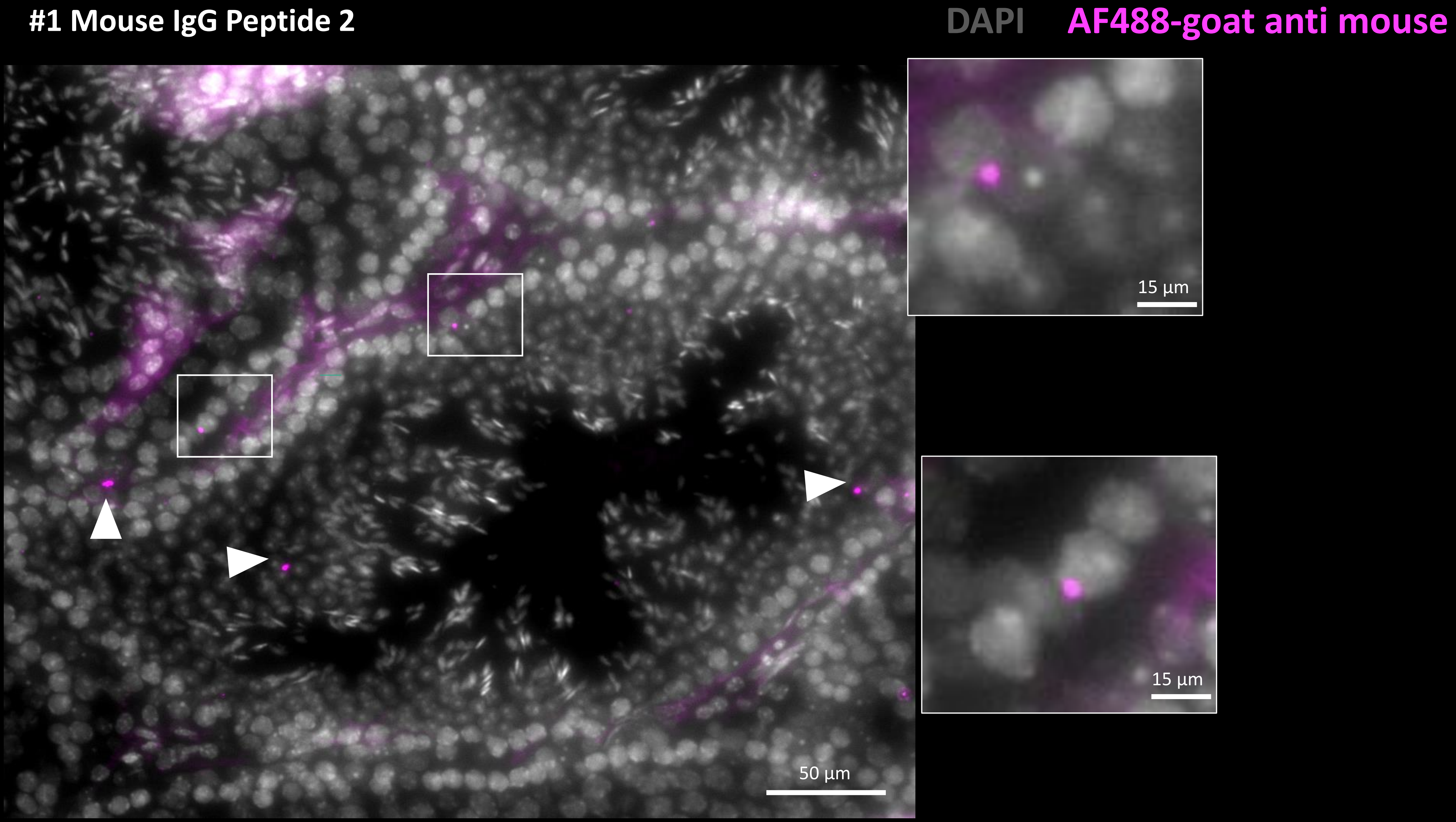

# #2 Mouse IgG Peptide 2

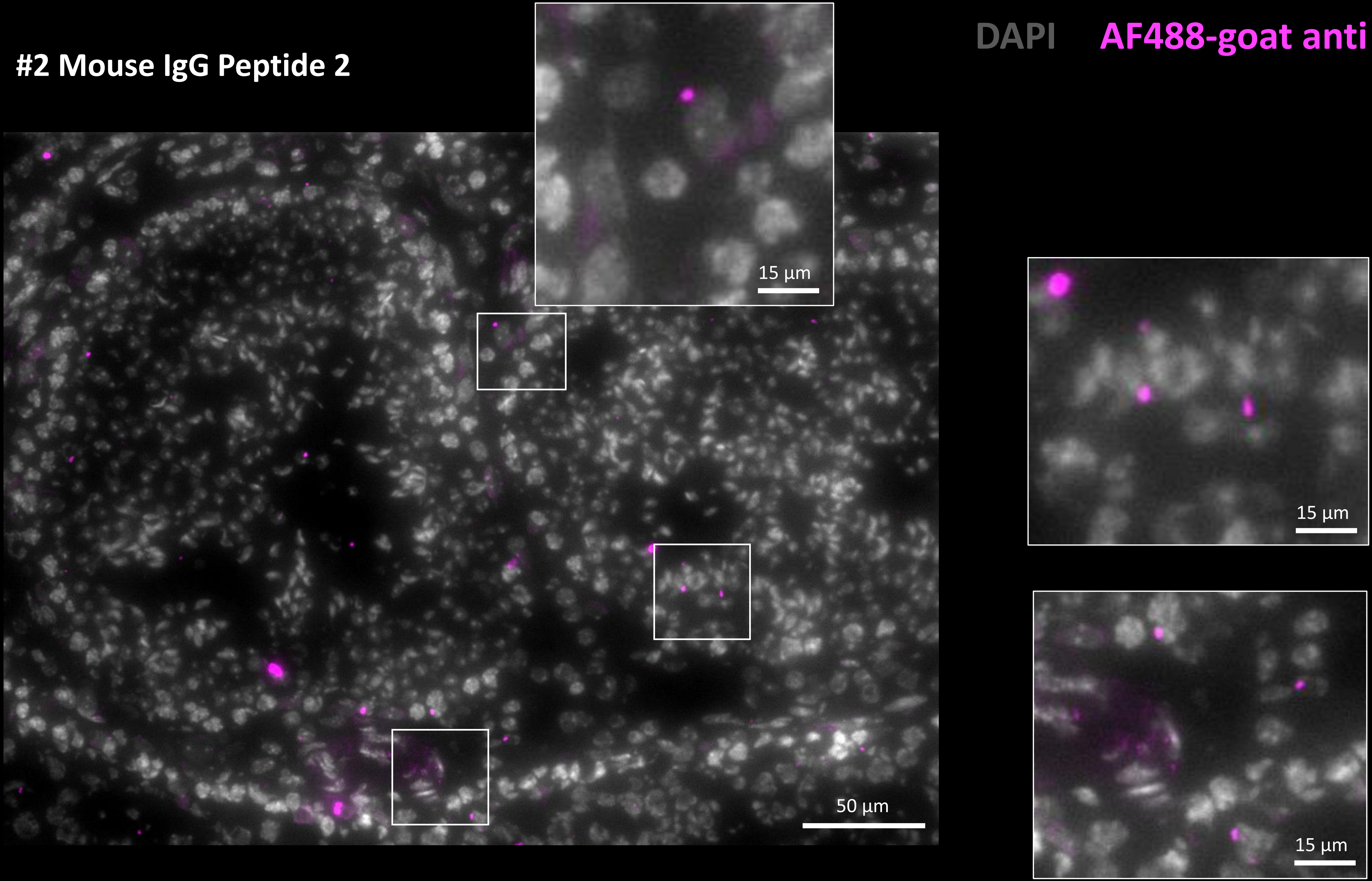

#3 Mouse IgG Peptide 2

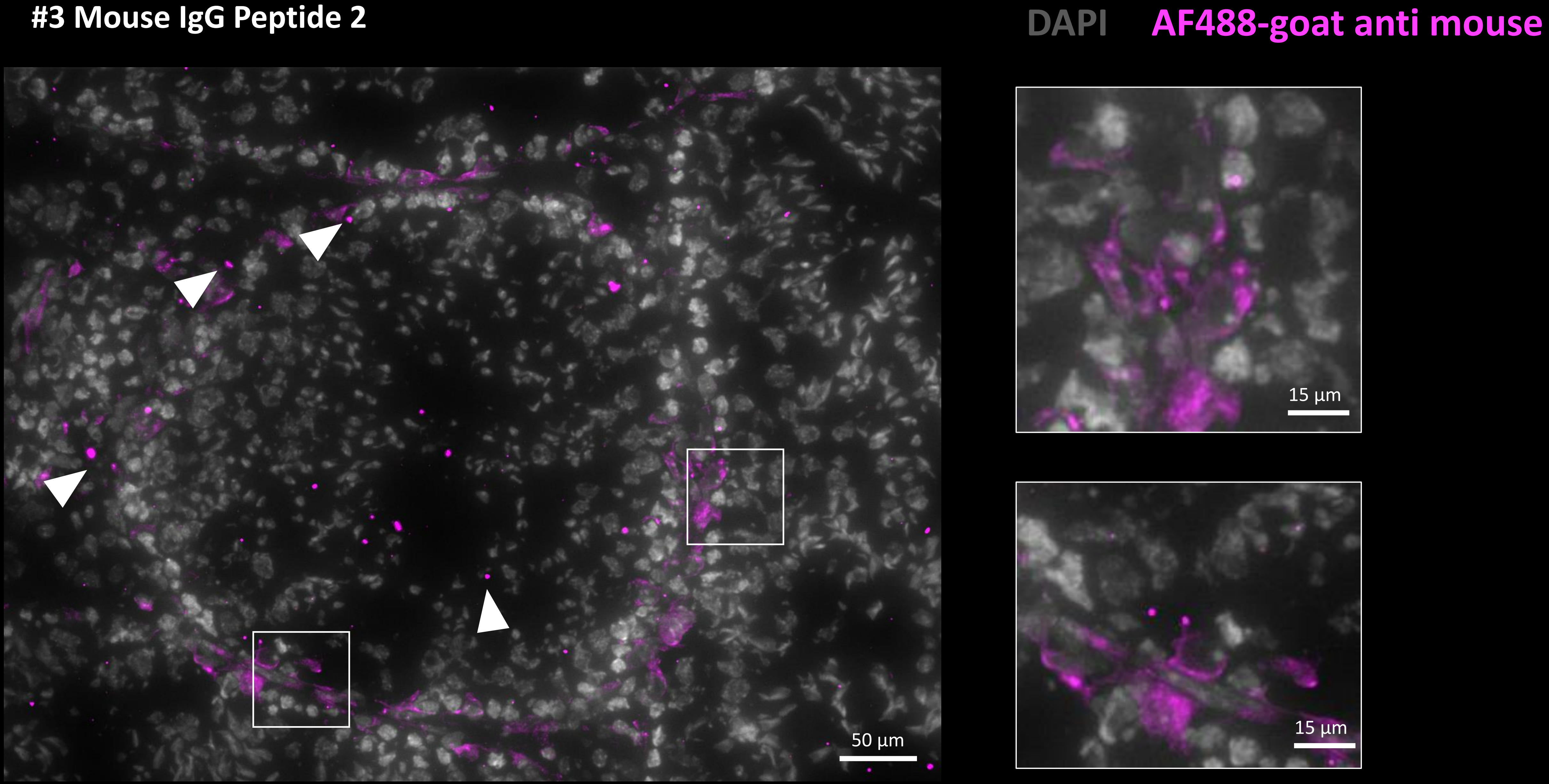

# #4 Mouse IgG Peptide 2

DAPI AF488-goat anti mouse

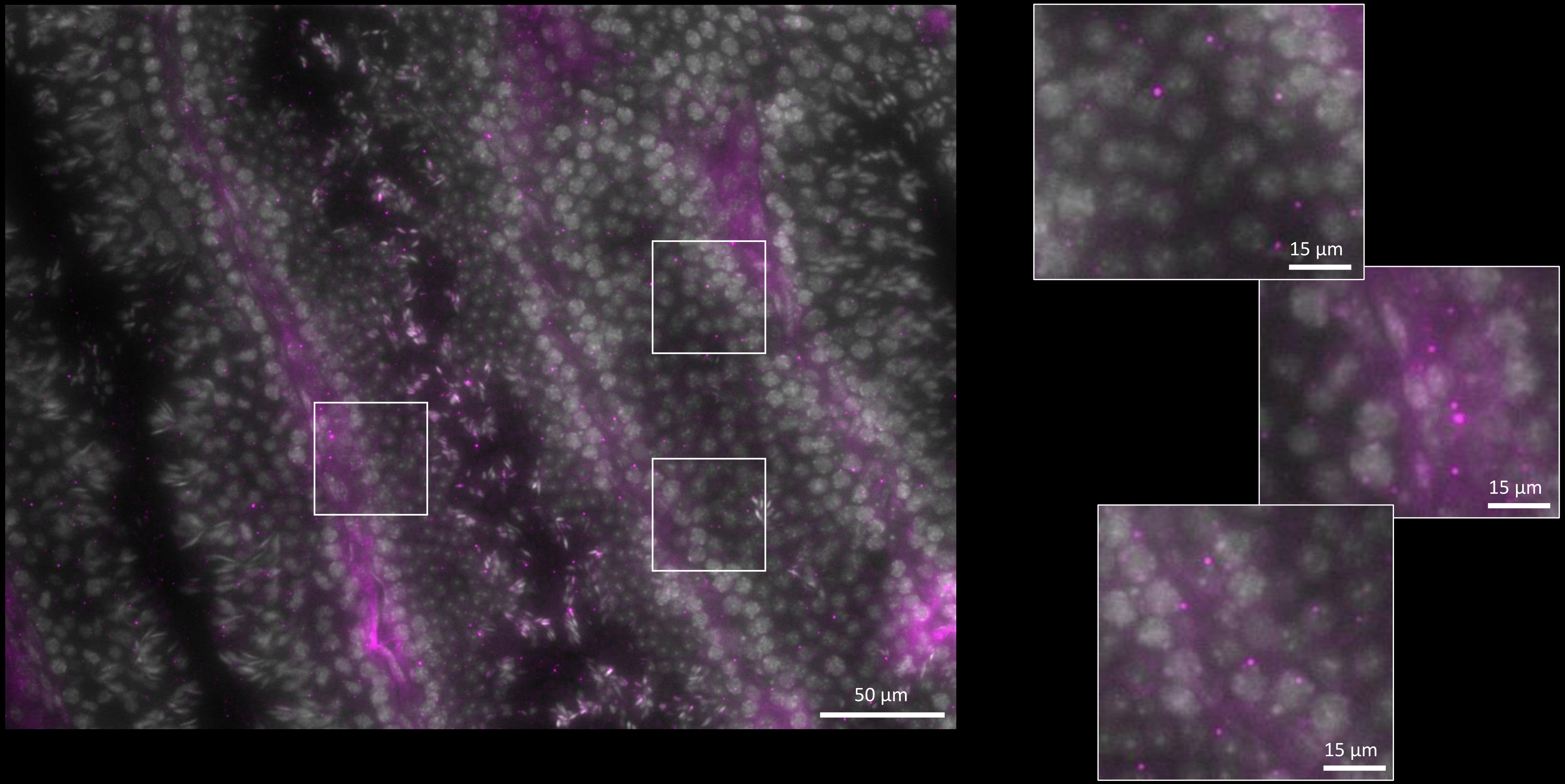

## #5 Mouse IgG Peptide 2

DAPI AF488-goat anti mouse

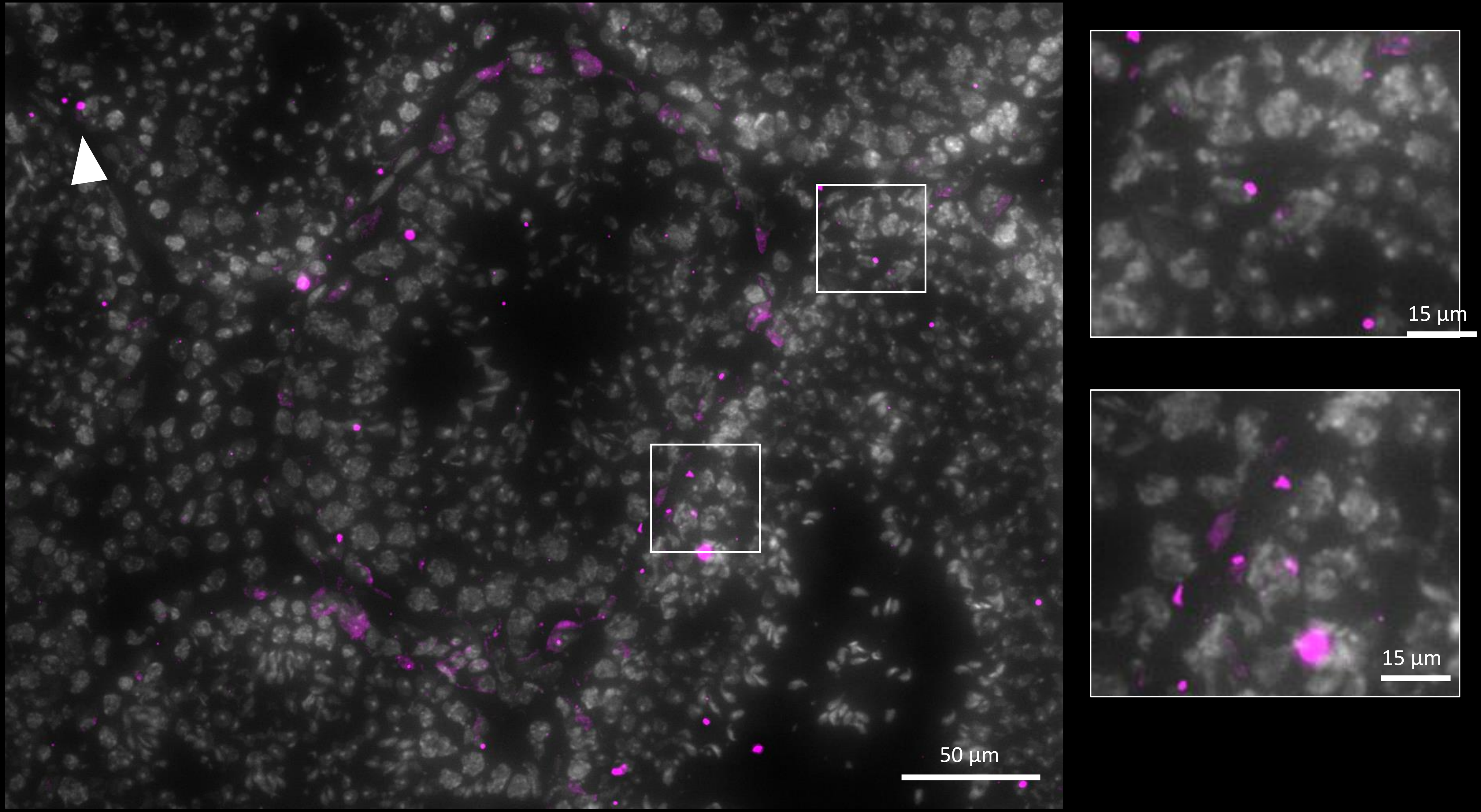

DAPI AF488-goat anti mouse

# #6 Mouse IgG control

Merge

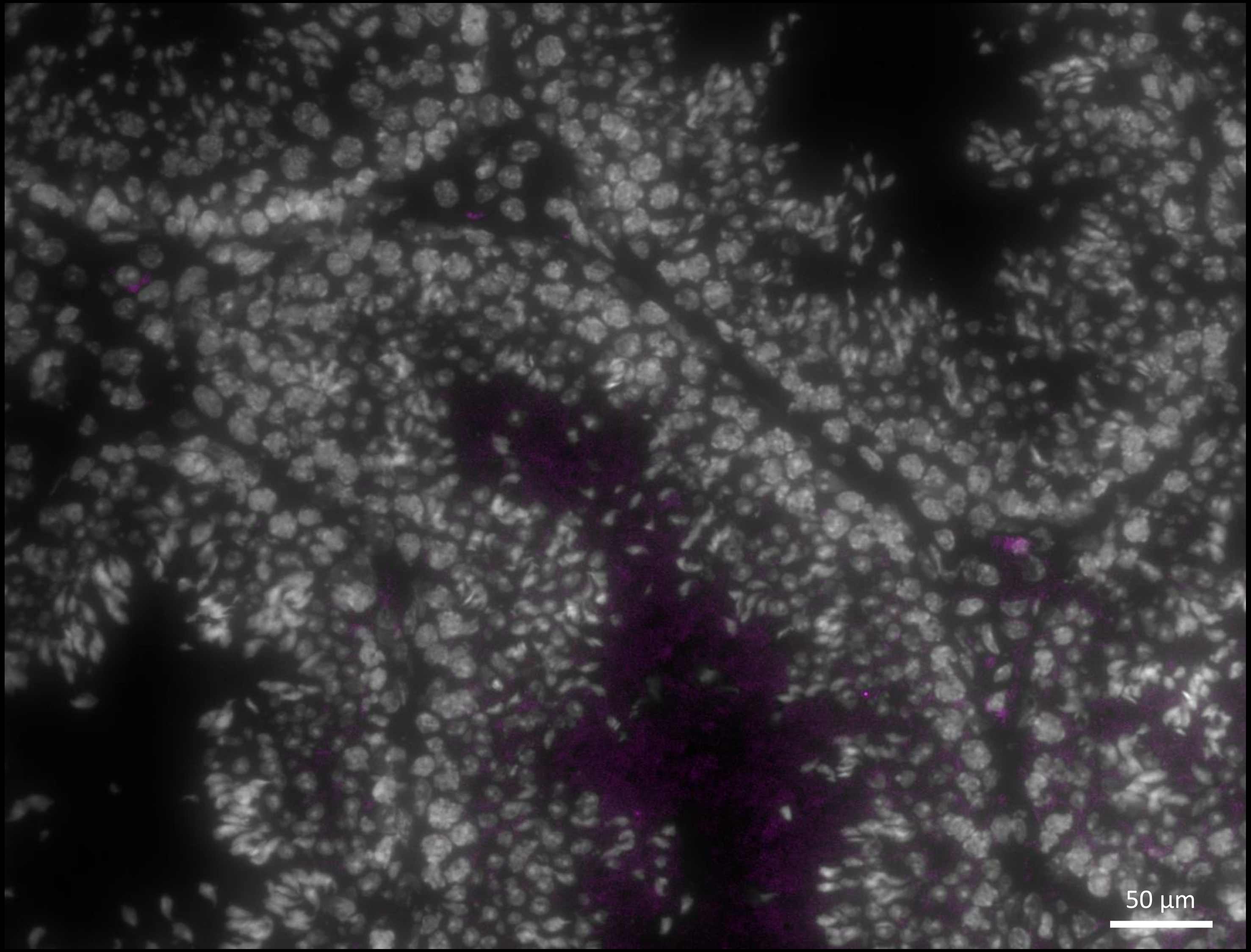


AF488-goat anti mouse

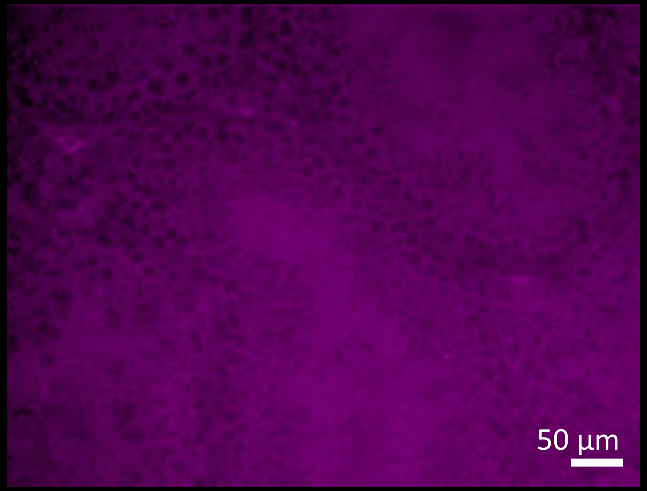

DAPI AF488-goat anti mouse

#7 Mouse IgG control

Merge

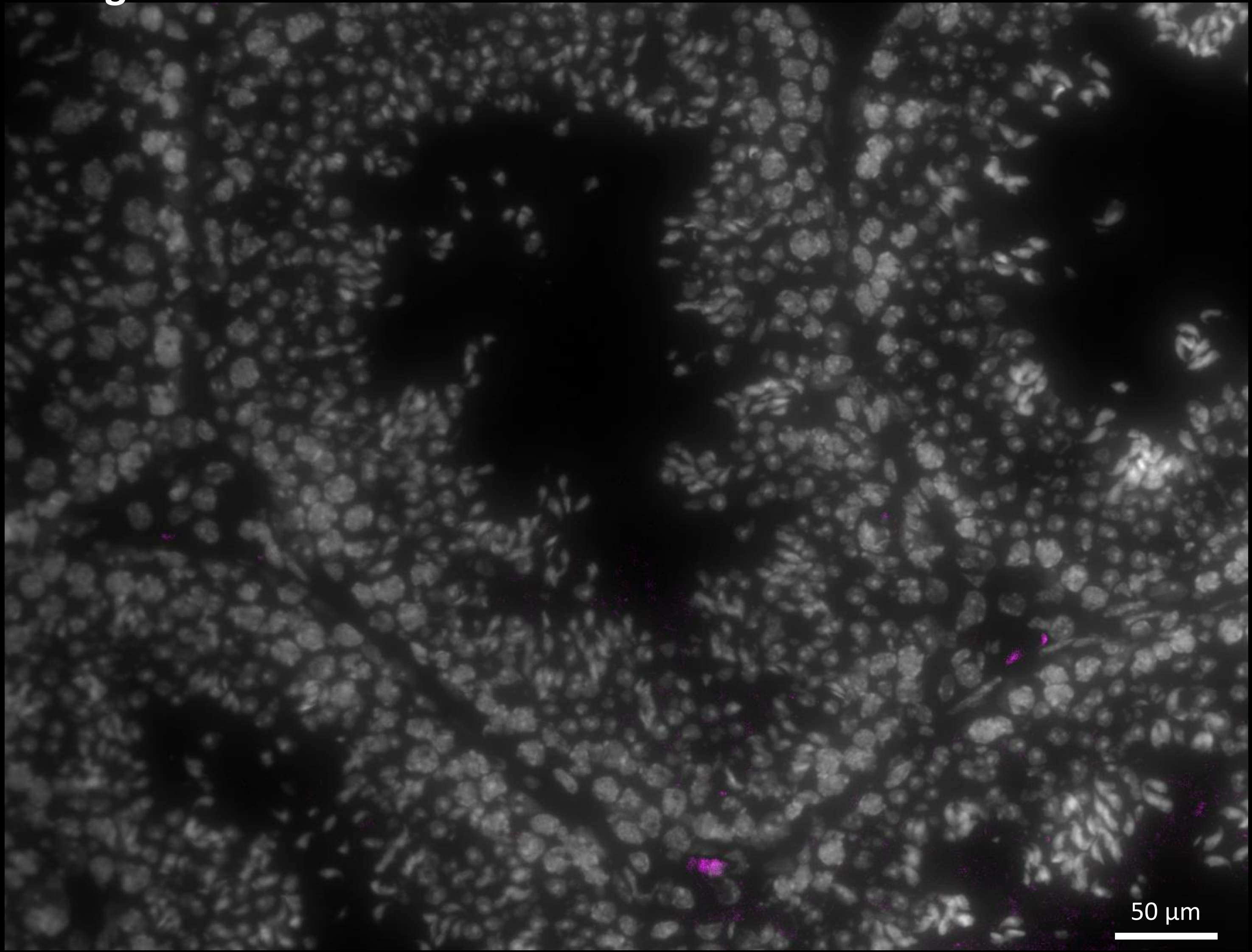


AF488-goat anti mouse

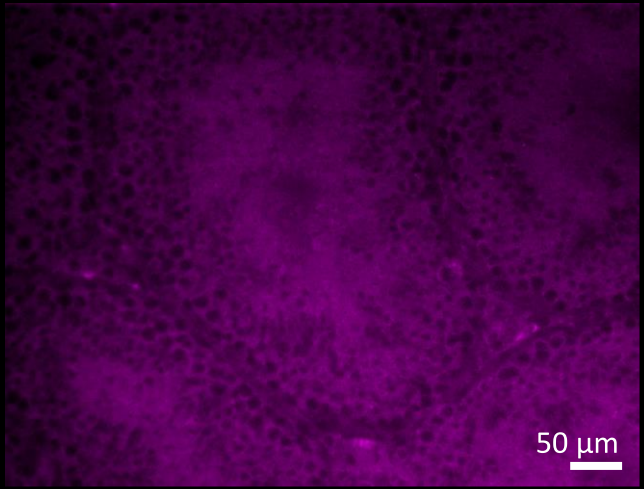

DAPI AF488-goat anti mouse

#8 only AF488

Merge

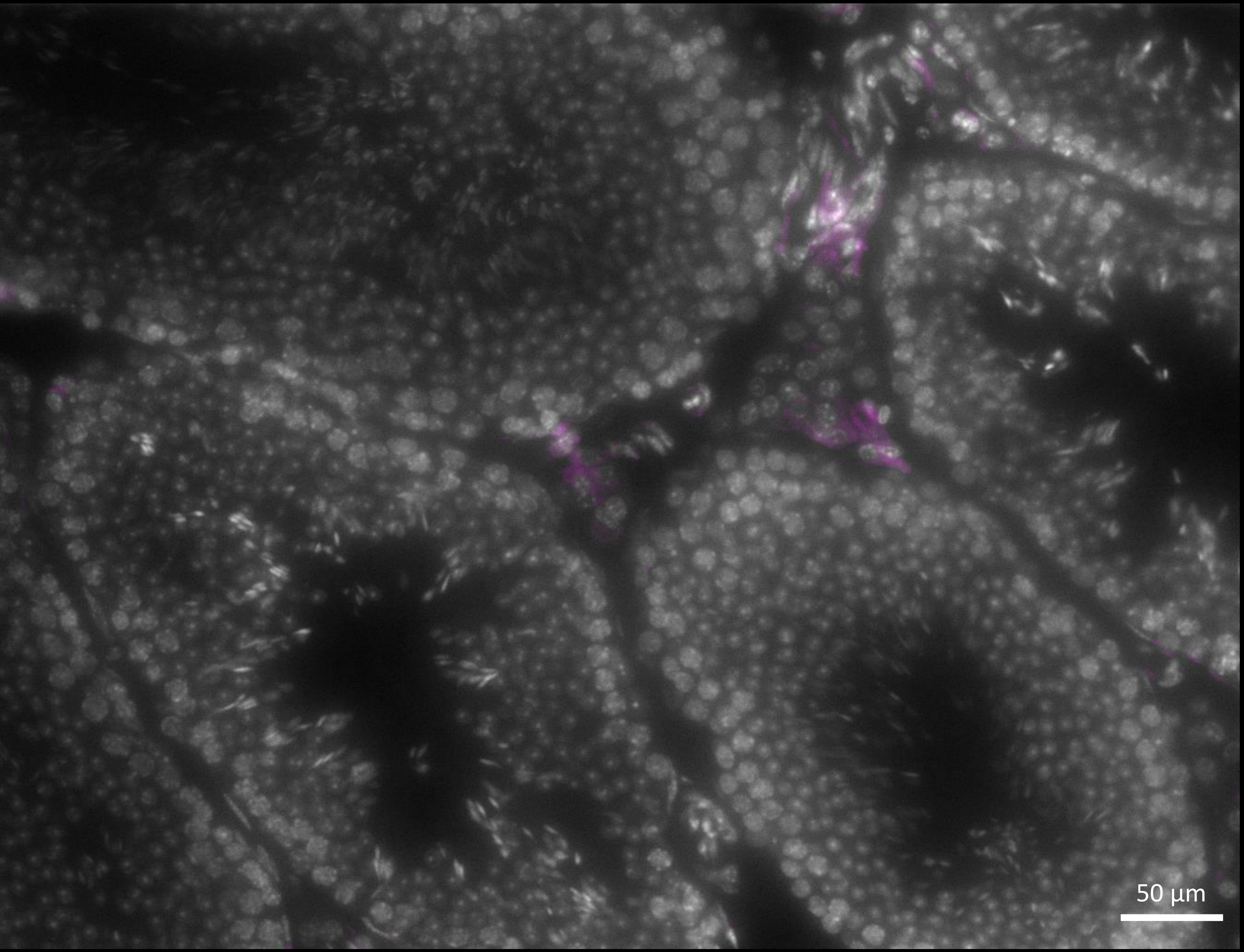


AF488-goat anti mouse

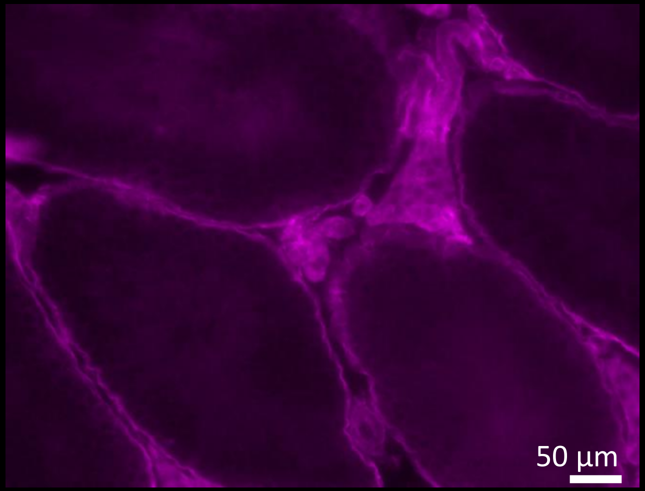

Supplement: Supplementary file 1 [file DataSheet1.pdf]
