# Supplementary figures and images for: Long COVID-19 autoantibodies and their potential effect on fertility
Source: Front Immunol. 2025 May 27;16:1540341. doi: 10.3389/fimmu.2025.1540341 (PMC12149208; doi:10.3389/fimmu.2025.1540341)

# Unedited blot and gel images

Full unedited gel for Figure 5A

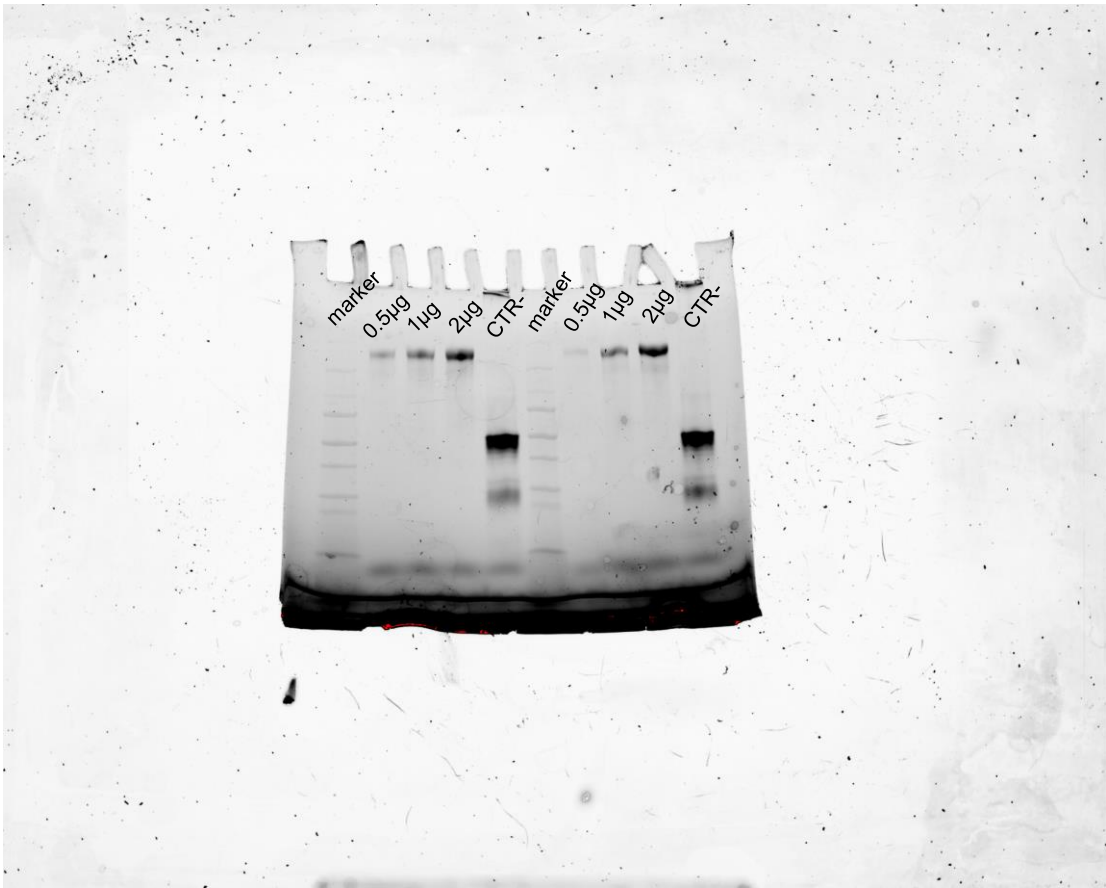

Full unedited blot for Figure 5A

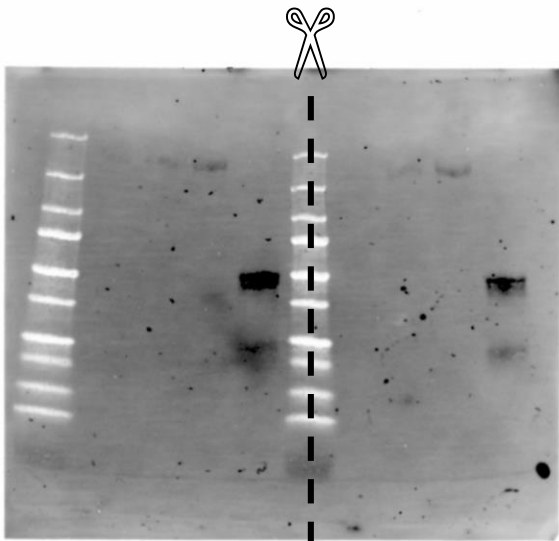

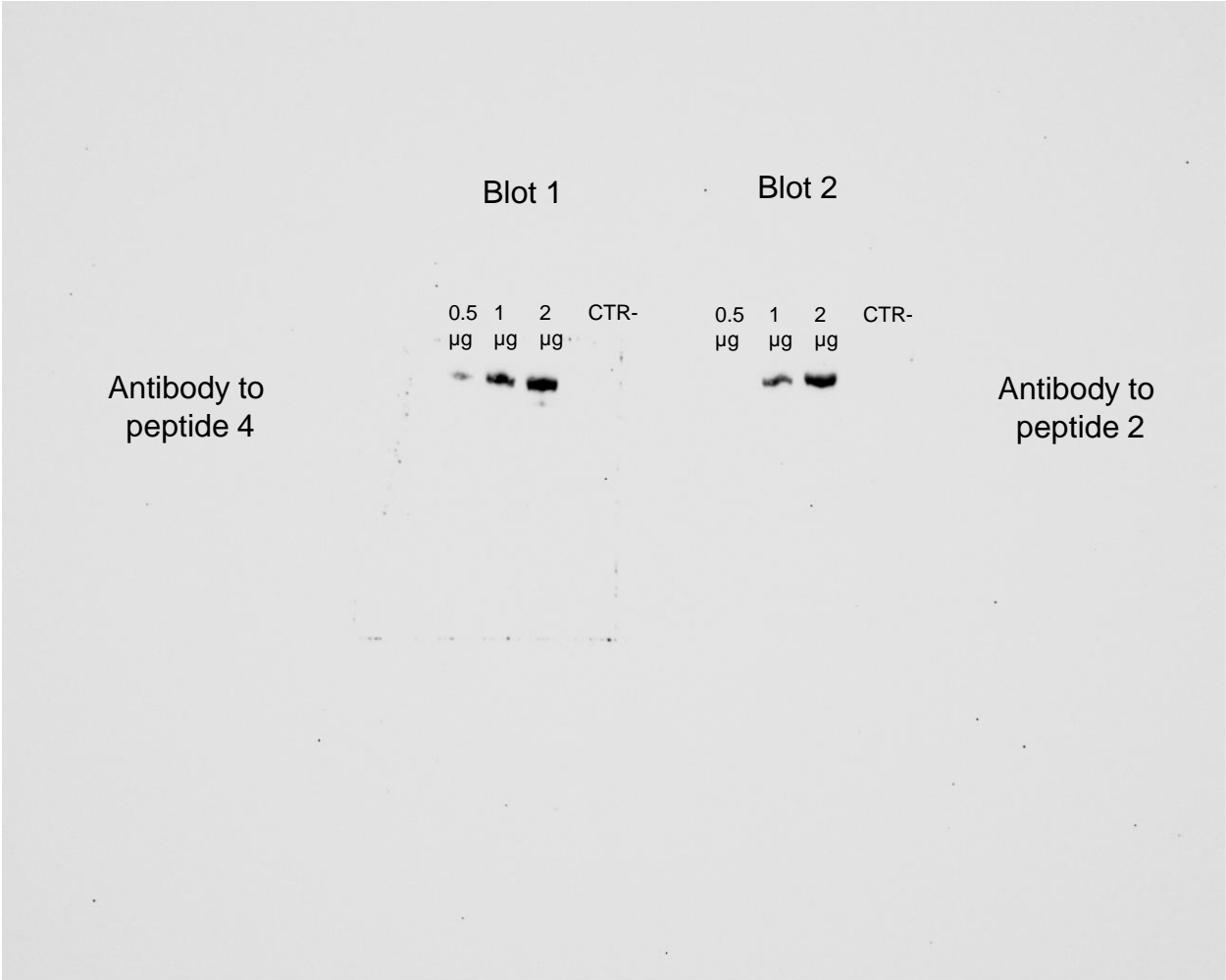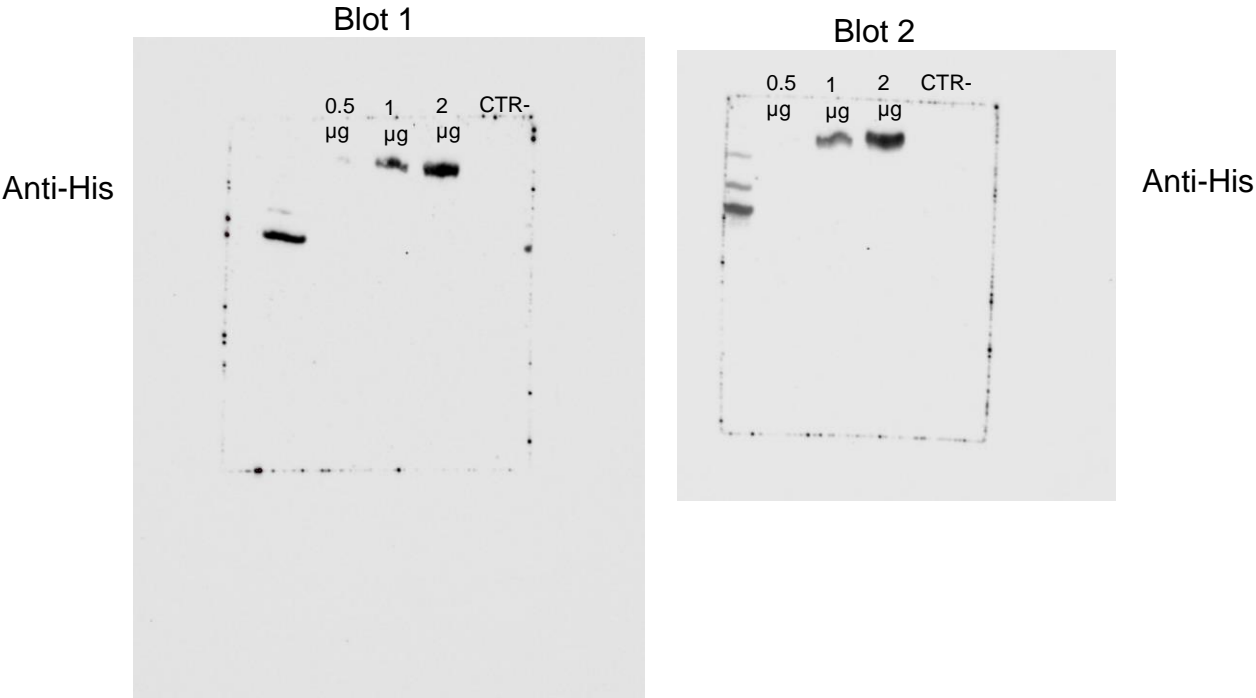

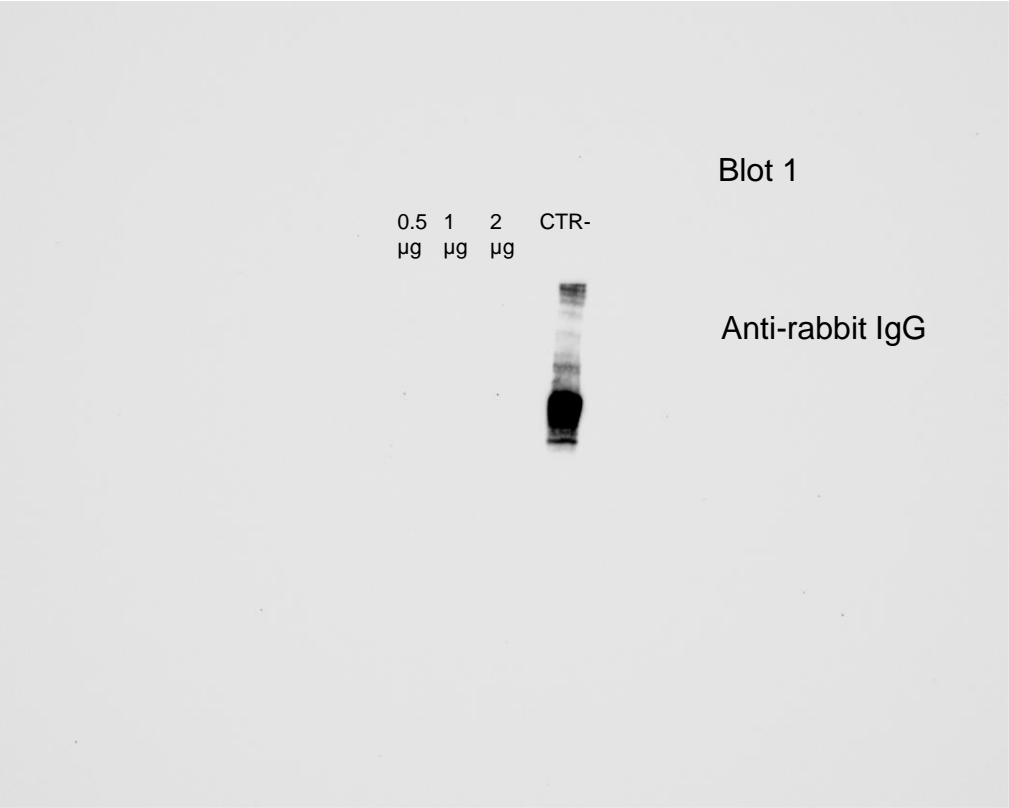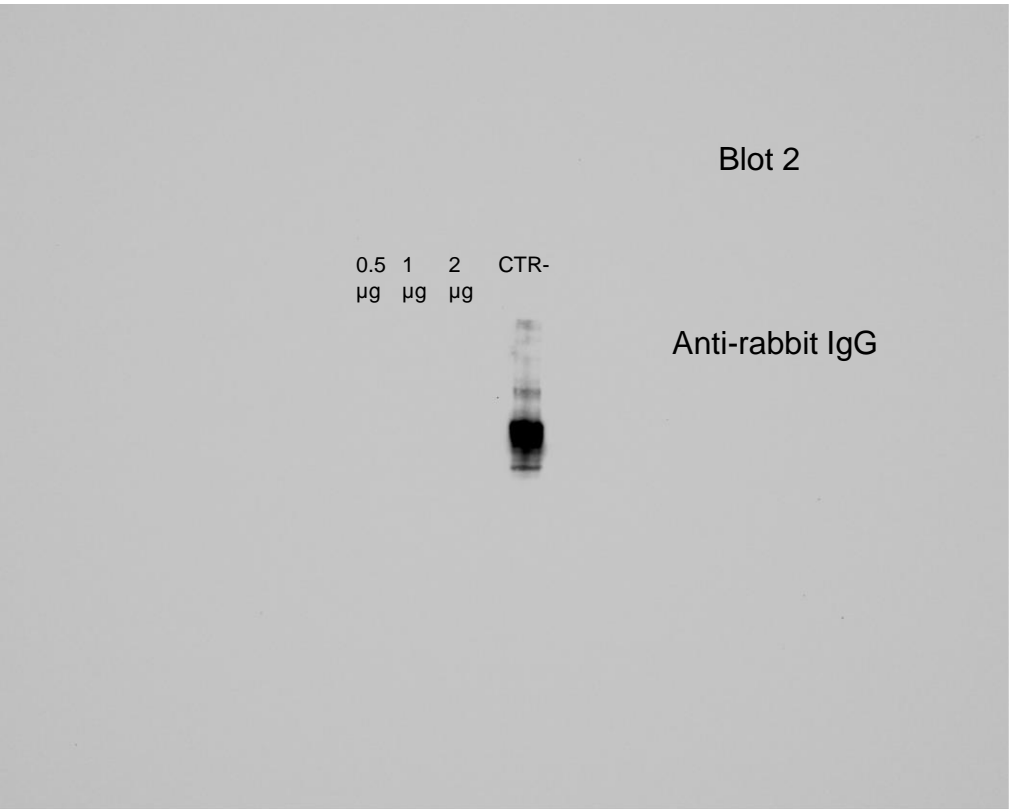

Supplement: Supplementary file 2 [file DataSheet2.pdf]
