# Supplementary material for: Long COVID-19 autoantibodies and their potential effect on fertility
Source: Front Immunol. 2025 May 27;16:1540341. doi: 10.3389/fimmu.2025.1540341 (PMC12149208; doi:10.3389/fimmu.2025.1540341)
Supplement: Supplementary file 3 [file Table1.docx]

Supplementary Material

**Long COVID-19 autoantibodies and their potential effect on fertility**

Laura Talamini, Dennyson Leandro M. Fonseca, Darja Kanduc, Olivier Chaloin, Cindy Verdot, Christian Galmiche, Arad Dotan, Igor Salerno Filgueiras, Maria Orietta Borghi, Pier Luigi Meroni, Natalia Y. Gavrilova, Varvara A. Ryabkova, Leonid P Churilov Gilad Halpert, Christian Lensch, Lorenz Thurner, Siew-Wai Fong, Lisa F.P. Ng, Laurent Rénia, Barnaby E. Young, David Chien Lye, José Manuel Lozano, Otávio Cabral-Marques, Yehuda Shoenfeld, Sylviane Muller

**Supplementary Table S1.** Characteristics of patients and control individuals evaluated in this study

A

**A**

| **Cohort** | **Age (years)** | **Gender** | |
| --- | --- | --- | --- |
|  | Average ± SD | Man (n) | Woman (n) |
| Acute COVID-19 (n= 87) | 53 ± 14.8 | 68 | 19 |
| Long COVID-19 (n= 76) | 42 ± 12.9 | 28 | 48 |

| **Country** | **Cohort** | **Age (years)** | **Gender** | |
| --- | --- | --- | --- | --- |
|  |  | Average ± SD | Man (n) | Woman (n) |
| Germany | Acute COVID-19 (n = 36) | 60.1 ± 12.1 | 30 | 6 |
|  | Long COVID-19 (n = 61) | 47.8 ± 12.6 | 24 | 37 |
| Russia | Long COVID-19 (n = 15) | 35.2 ± 10.9 | 4 | 11 |
| Singapore | Acute COVID-19 (n = 51) | 45.9 ± 13.6 | 38 | 13 |
| France | Non-COVID-19 (n = 19) | NA | NA | NA |

**B**

NA, not applicable (voluntary, non-remunerated blood donors)

**Supplementary Table S2.** List of 667 spermatogenesis-related proteins (given by Uniprot entry).

AFG2H; SPAT7; SUN5; SPE39; SOLH1; SPA24; M1AP; SPAT2; GMCL1; SPAT8; TOPK; SPC1L; SPT13; ESX1; GPAT4; MIEAP; TX101; SPAT6; SPT16; DJB13; ALKB7; S31E1; SOLH2; SPT46; SPT20; SPT22; SPT25; SPAT4; SPAT9; S31A7; SPT32; SPT12; SPT19; SPT21; S31C1; SPA5L; SPAS2; SPERI; S31A1; SPA6L; S31C2; SPAS1; PIWL1; BRDT; S31A3; SYCP3; SPAG4; NR2C2; PSME4; CREM; CDYL; ACBG2; S31A6; SPT33; S31A5; PSMA8; TSSK2; TDRD9; RBY1A; SEP12; DAZ2; IFT20; HSF2B; SPEF2; AURKC; ASF1B; CDK16; DAZ1; S31D4; PRM2; S31D3; S31D1; TM203; TSSK1; TCFL5; TDRD7; UT14C; TSG10; RGAP1; SMBT1; TDRKH; TSNAX; PIWL4; PACR; OAZ3; SIAH1; ZCPW1; USP9Y; ASZ1; NAL14; LGR4; PLD6; MTA70; MAK; PIWL2; PAX5; MET14; RHBL4; RNF8; RBY1F; PUM1; TEX14; SPAG8; RN114; SMRP1; BNC1; CALR3; CHD5; ARI4A; ALKB5; FKBP6; DAZL; BOLL; FOXA3; HSP72; LARP7; GMCL2; DZIP1; GTR14; BAG6; CC136; DDX4; CADM1; DAZP1; PCH2; ADA18; DAZ3; DAZ4; GGN; TM119; MAEL; SEPT4; TSSK3; STP1; STRBP; TESK1; SUN1; YTDC2; S22AG; TCP11; ODFP2; TAF4B; SEPT7; TBP; TRXR3; WDR33; TT21A; UBR2; SYNE1; TBC21; STK11; TXND3; TSPY1; TESK2; ODFP4; MYBA; S26A8; SETX; SL9C1; RARA; TYY1; SEPT2; SEPT6; GORS2; PAR11; M10L1; H2B1A; MYCBP; KHDR3; NPHP1; NANO3; RFX2; ODFP1; ADCYA; BRCA2; CDAC1; BRD2; IHO1; KHDR1; ARI4B; DS13B; CTSR1; ADA29; FANK1; DHX36; FSHB; JAM3; LSHB; KDM5D; MFA3L; KDM3A; FSHR; IFT27; CFA69; CP131; DNM3L; CFA91; RAI14; H1T; SO4C1; SPA2L; HSP1; ZN318; HORM1; HILS1; NANO2; SPAT1; TXND2; UBP42; PMFBP; HERC4; NPAP1; PDILT; IFT25; STRA8; TXND8; D19L2; TDRD1; RN151; FSIP2; TYRO3; TWSG1; SYCP1; SSR1; TEST; PKHA1; ROS1; ZNT9; UFO; ZNRF4; ZDHC3; RUVB1; SCMH1; SSR2; SPO11; SRSF3; SMAD4; SOX9; TRI27; RA51C; SCF; SOX17; SIR1; TAF1L; TSSK4; UBA6; SYCE1; UNC50; PSA7; RU2A; SKAP; TDR12; TEX15; TBD; RNF32; SPZ1; THEG; RHF2B; ZN296; VCX1; TEX19; VCX2; ZPBP1; RD21L; RN141; TSN8; TSPY2; ZN383; TDRP; VCY1; TIP39; VCX3; ZN541; TF2AY; RBY1C; ZMY15; SPT45; TSPY8; TOPZ1; SCML1; TAF7L; ZN200; SAM13; S6OS1; TBATA; RNF17; ZSCA2; SGPL1; STIM1; TDRD6; UB2R1; UBE2B; WFDC2; YBOX2; SKIL; YBOX3; SODC; TPPP2; TSKS; SHP1L; TDRD5; WIPF3; TXIP1; WDR48; ST3L3; RBMX; TSSK6; SYCE3; ZN331; TSPY3; VCY2; ZGLP1; MEIOS; MORC1; MNS1; MTMR5; PRS42; PC11Y; PCY1B; PSB4; MEI4; PSB1; PSB3; PSB6; DYRK2; PGK2; TRI69; TPGS1; TSPY4; TSPYA; TALD3; TAF1; SPTA3; XRN2; REC8; SSR3; TRA2B; SMS1; SSRB; STP2; STF1; ZNF35; VCX3B; PIWL3; LRGUK; PROF3; RBY1D; QRIC2; SHSA6; SOX8; RBY1E; RGN; PRM3; SPEM1; ZFP41; YA037; AT2B4; IF2GL; ACOX1; ADIG; B4GN1; CNR1; DYSF; CXB3; DAF; DNJA1; HERC2; IFT81; GGT5; IFTAP; INCA1; CIP2A; ADAD1; GLTL5; PCDG4; P63; ING2; FAM9A; KIT; MSH5; PA1B3; PSA6; MEIOB; PHC2; F166A; HOOK1; I20L2; G3ST1; HSF1; IF2G; KAD7; MEA1; MYBPP; GGNB1; MGT4D; HXA10; IF4E; IF122; MCH; MOS; MTL5; NDC1; KAPCG; MPIP3; NOTC1; NANO1; NUP62; HSFY1; KDM5A; OVOL1; GGNB2; H31; HXA9; AT2A2; NDRG3; OXLA; PDGFA; PIAS1; LRC18; FA50A; IF5A2; K1C9; PSA1; PSA4; PTTG1; P2R3C; PSB5; GPX4; MERTK; NACA; ODF3A; PSMD6; SPNXA; RIM3B; SG11B; SUN3; PAIP2; MCSP; MORN2; MRO2B; MPRI; OR7C1; NJMU; LCORL; MCMD2; NSUN2; PROK2; RBY1B; PRDX4; LRRK2; MSH4; NR0B1; LRWD1; PRY; PSA5; PRKRA; PSB7; RIM3A; PATZ1; LYRM9; RIM3C; RHXF1; PHF7; PGAM2; RN138; STON1; PO4F2; PSA2; PSA3; RGS2; TEX11; SACA1; ROP1B; PA1B2; SPT48; SP17; POC1A; RL39L; SUV92; STYX; AKA11; BMAL1; CABS1; BTBDI; CF299; BEX1; BAP31; AGRG2; AZIN2; CCNY; B2CL2; CF251; ATS2; BTG1; CCD63; ACTT1; B3A2; CDK1; CECR2; ASAH1; ACTZ; B2L11; EXD1; EZHIP; H31T; I5P2; JAG2; ERCC1; HOATZ; AAKG1; AMZ2; FND3A; GAMT; H2AB2; DYDC1; EFCB9; DYLT2; ACE; H3Y2; ADA10; CEP19; CTSRD; AKAP4; CALR; AGM1; AT1A4; DYN2; CCD91; DYH1; AP3B1; DEDD; DKKL1; CTSRZ; CTSRB; CYLC1; ASPM; CCNB1; DMC1; CDY2; FAM9B; CTSR3; CTSRG; FAM9C; CCD87; GGT3; CATL2; CLOCK; DRC9; BCL6; CELF3; DNM3A; CPSM; KATL1; H33; MICLK; GHSR; HSF2; JAM2; ISK2; ITPR3; MLH1; FXR1; GTSF1; LDHC; NKAPL; NDK5; HEMGN; LIMK2; H3PS2; IQCH; MEIG1; MRAS1; H3C; H3Y1; G3PT; GGT7; ANR49; ARMC2; GGT1; KASH5; H11; HERP2; KLH10; FOXJ1; H32; INSL3; IPSP; KIFC1; ACTL9; APOB; IMP2L; H2AX; HXA11; KDM2B; E2F1; ATAT; CCYL1; CLGN; BRME1; ADA28; GLI1; FSIP1; GOGA3; H2AB1; CTSR4; CCD89; CTSR2; DMRT1; ELL3; CALI; CFA54; CELF1; BAD; CCNI; CIB1; DDX25; FA71B; FANCM; CFA44; CRTAP; ASF1A; CSK22; DB118; FACOS; H1FNT; ANDR; CF157; DJC15; F181A; GGT2; DPCD; FHL5; FSTL3; CNTD1; ATRX; CLD11; CYLC2; CFA43; CCNA1; CRKL; CST8; AGFG1; CAMP; CP26B; CR3L4; CDY1; BIRC3; B2CL1; CCD83; CETN2; CXA1; CL054; AVR2A; B2L10; CNBD2; CRIS2; RD23B; RPGR; SPT17; RHXF2; ODPAT; PLAT1; ROP1A; RL10L; PBX4; PSB2; RNFT1

**Supplementary Table S3.** List of 217 spermatogenesis-related proteins sharing pentapeptides with SARS-CoV-2 Spike protein (given by Uniprot entry)

AAKG1; ACBG2; ACTL9; ACTT1; ADA10; ADA18; ADA29; ADAD1; AGFG1; AGRG2; ALKB5; ANDR; AP3B1; APOB; ARI4B; ASPM; AT2B4; ATRX; ATS2; B2CL1; BCL6; BNC1; BRCA2; BRD2; BRDT; CABS1; CALI; CATL2; CC136; CCD87; CCD91; CCYL1; CF251; CFA43; CFA54; CFA91; CHD5; CIP2A; CLD11; CLOCK; CP131; CREM; CRTAP; CTSR1; CTSR3; CTSRB; CTSRD; CXA1; DMC1; DMRT1; DYH1; DYN2; DYSF; ERCC1; FA71B; FANCM; FANK1; FND3A; FSIP1; FSIP2; FXR1; GGNB2; GGT5; GGT6; GOGA3; GPAT4; GTR14; GTSF1; HERC2; HERC4; HILS1; HSF1; HSP72; HSP72; IF122; IF4E; IFT20; IHO1; INCA1; IQCH; ITPR3; JAG2; JAM3; KASH5; KIFC1; KLH10; LCORL; LGR4; LRRK2; LSHB; M10L1; M1AP; MAK; MCMD2; MEIOB; MFA3L; MGT4D; MOS; MPRI; MRO2B; MSH4; MSH5; MTA70; MTL5; MTMR5; MYBPP; NAL14; NDC1; NJMU; NOTC1; NPHP1; NR0B1; NR2C2; NUP62; OR7C1; PA1B3; PACR; PAIP2; PC11Y; PDILT; PIAS1; PIWL3; PRKRA; PROK2; PSB1; PSME4; QRIC2; RD21L; REC8; RFX2; RGAP1; RIM3A; RIM3B; RIM3C; RNF17; ROS1; S26A8; S31A5; S31A7; S6OS1; SCF; SCMH1; SCML1; SEP12; SEPT6; SETX; SKIL; SL9C1; SMBT1; SOLH1; SOX17; SPA2L; SPAS1; SPAS2; SPAT1; SPAT4; SPAT7; SPAT9; SPEF2; SPERI; SPT13; SPT21; SPT32; SPT48; SPZ1; SSR1; SSR2; STIM1; SUN1; SYCP1; SYNE1; TAF1L; TAF7L; TALD3; TBC21; TCFL5; TCFL5; TDRD1; TDRD5; TDRD6; TDRD9; TESK1; TESK2; TEX14; TF2AY; THEG; TOPK; TOPZ1; TRI27; TRI69; TSKS; TSSK1; TXND3; TYY1; UB2R1; UBA6; UBE2B; UFO; UNC50; USP9Y; UT14C; VCX1; VCX2; VCX3; VCX3B; VCY1; WIPF3; WIPF3; XRN2; YTDC2; ZMY15; ZN296; ZN318; ZN331; ZNF35; ZPBP1; ZSCA2

**Supplementary Table S4.** Peptide sharing between SARS-CoV-2 Spike protein and spermatogenesis-related proteins.

| **Shared**  **Peptides** | **Spermatogenesis-associated protein and functions/pathologies*** | **Ref** |
| --- | --- | --- |
| LGDIA | *SPT48. Spermatogenesis-associated protein 48*  **Essential for spermatogenesis** | (1) |
| GAGAA,  DEDDS | *ANDR. Androgen receptor*  Causes resistance to androgen and can lead to under virilized or infertile males | (2–4) |
| LDPLS,  VTTEI | *HILS1. Spermatid-specific linker histone H1-like protein*  Regulates gene transcription during mammalian spermiogenesis | (5) |
| LTPGD | *SPAS1. Spermatogenesis-associated serine-rich protein 1*  Has a role in the initiation of the first spermatogenic wave, and in the establishment or progression of the first male meiotic division | (6) |
| ASALG | *SOLH1. Spermatogenesis- and oogenesis-specific basic helix-loop-helix-containing protein1*  Transcription regulator of both male and female germline differentiation. Relates to non-obstructive azoospermia | (7) |
| GLTGT | *PIWL3. Piwi-like protein 3*  Spermatogenic failure | (8) |
| GTNGT, YRVVV | *SPAS2. Spermatogenesis-associated serine-rich protein 2*  Plays a role in testicular germ cell development | (9) |
| RVYSS | *S31A7. Spermatogenesis-associated protein 31A7*  Lower expression of AEP1 was observed in human testes diagnosed with hypospermatogenesis | (10) |
| LPLVS | *SPAT4. Spermatogenesis-associated protein 4*  Infertility | (11) |
| QDVVN  DVVNQ | *GTSF1. Gametocyte-specific factor 1*  Required for spermatogenesis | (12,13) |
| LGFIA | *S26A8. Testis anion transporter 1*  Relates to primary infertility, sperm morphologic abnormalities, asthenozoospermia characterized by a percentage of progressively motile sperm abnormally low. | (14) |
| AGSTP  ILSRL | *TESK2. Dual specificity testis-specific protein kinase 2*  predominantly expressed in testis | (15) |
| VTQQL | *ZPBP1. Zona pellucida-binding protein 1 precursor*  Implicated in sperm-oocyte interaction during fertilization | (16) |
| SEPVL | *SPAT9. Spermatogenesis-associated protein 9*  An important factor in male infertility | (17) |
| PSKPS | *TSSK1. Testis-specific serine/threonine-protein kinase 1*  Essential for male fertility | (18) |
| AQKFN | *SPZ1. Spermatogenic leucine zipper protein 1*  It may play an important role in spermatogenesis in the humans | (19) |
| ASTEK | *SPEF2. Sperm flagellar protein 2*  Required for correct axoneme development in spermatozoa. Essential for male fertility | (20) |
| KNKCV | *GGNB2. Gametogenetin-binding protein 2*  Necessary for testis morphology and sperm development | (21) |
| LEPLV,  ANLAA | *YTDC2. 3'-5' RNA helicase YTHDC2*  Required for both spermatogenesis and oogenesis. Essential for a successful meiotic program in the mammalian germline | (22) |
| PGQTG | *DMRT1. Doublesex- and mab-3-related transcription factor 1*  Key role in male sex determination by controlling testis development. Plays a key role in postnatal sex maintenance by maintaining testis determination and preventing feminization | (23–26) |
| SFIED | *SPATA7. Spermatogenesis-associated protein 7*  Expressed in primary spermatocytes in early prophase of meiosis I | (27) |

* Details and further references for functions/pathologies from PubMed, Online Mendelian Inheritance in Man (OMIM) and UniProt public databases

**Supplementary Table S5.** Distribution of pentapeptides shared between SARS-CoV-2 Spike protein and spermatogenesis-related proteins among experimentally validated SARS-CoV-2 Spike protein-derived epitopes

| **IEDB ID** | **Epitopes** |
| --- | --- |
| 51999 | QPYRVVVLSF |
| 57592 | SEPVLKGVKL |
| 1072965 | SVTTEILPVSMTKTS |
| 1074838 | AEIRASANLAATK |
| 1074866 | CALDPLSETK |
| 1074898 | FTISVTTEIL |
| 1074918 | GYQPYRVVVLSF |
| 1074928 | ILPDPSKPSK |
| 1074967 | LEPLVDLPI |
| 1074989 | LSSTASALGK |
| 1075039 | RQIAPGQTGKIADYNYKL |
| 1075066 | SVLNDILSRL |
| 1075093 | VLPFNDGVYFASTEK |
| 1309118 | GPKKSTNLVKNKCVN |
| 1309132 | NFSQILPDPSKPSKR |
| 1309418 | AEIRASANLAATKMSECVLG |
| 1309447 | DFGGFNFSQILPDPSKPSKR |
| 1309467 | FDEDDSEPVLKGVKLHYT |
| 1309468 | FERDISTEIYQAGSTPCNGV |
| 1309475 | GCCSCGSCCKFDEDDSEPVL |
| 1309482 | GYQPYRVVVLSFELLHAPAT |
| 1309515 | LHRSYLTPGDSSSGWTAGAA |
| 1309519 | LPDPSKPSKRSFIEDLLFNK |
| 1309523 | LSSNFGAISSVLNDILSRLD |
| 1309531 | NGLTGTGVLTESNKKFLPFQ |
| 1309534 | NITRFQTLLALHRSYLTPGD |
| 1309554 | QAGSTPCNGVEGFNCYFPLQ |
| 1309567 | RDLPQGFSALEPLVDLPIGI |
| 1309589 | SYGFQPTNGVGYQPYRVVVL |
| 1309593 | TITDAVDCALDPLSETKCTL |
| 1309595 | TNFTISVTTEILPVSMTKTS |
| 1309600 | TYVTQQLIRAAEIRASANLA |
| 1309602 | VCGPKKSTNLVKNKCVNFNF |
| 1309603 | VKNKCVNFNFNGLTGTGVLT |
| 1309604 | VLNDILSRLDKVEAEVQIDR |
| 1309606 | VTLADAGFIKQYGDCLGDIA |
| 1309611 | WFHAIHVSGTNGTKRFDNPV |
| 1309616 | YEQYIKWPWYIWLGFIAGLI |
| 1309624 | YYHKNNKSWMESEFRVYSSA |
| 1310303 | CAQKFNGLTVLPPLL |
| 1310360 | EIYQAGSTPCNGVEG |
| 1310434 | GAISSVLNDILSRLD |
| 1310448 | GKLQDVVNQNAQALN |
| 1310592 | LLALHRSYLTPGDSS |
| 1310609 | LPDPSKPSKRSFIED |
| 1310612 | LPQGFSALEPLVDLP |
| 1310747 | QPYRVVVLSFELLHA |
| 1310785 | SALEPLVDLPIGINI |
| 1310855 | TNFTISVTTEILPVS |
| 1310899 | VLLPLVSSQCVNLTT |
| 1310947 | WTFGAGAALQIPFAM |
| 1311657 | CCSCGSCCKFDEDDSEPVLKGVKL |
| 1311782 | PDPSKPSKRSFIEDLLFNKVTLAD |
| 1314425 | ALDPLSETK |
| 1316945 | FSQILPDPSKPSKRSFIE |
| 1317060 | FTISVTTEI |
| 1323467 | QPYRVVVL |
| 1323750 | RASANLAATK |

**^1^** Epitopes listed as IEDB ID

**^2^** Shared peptides given in red letters


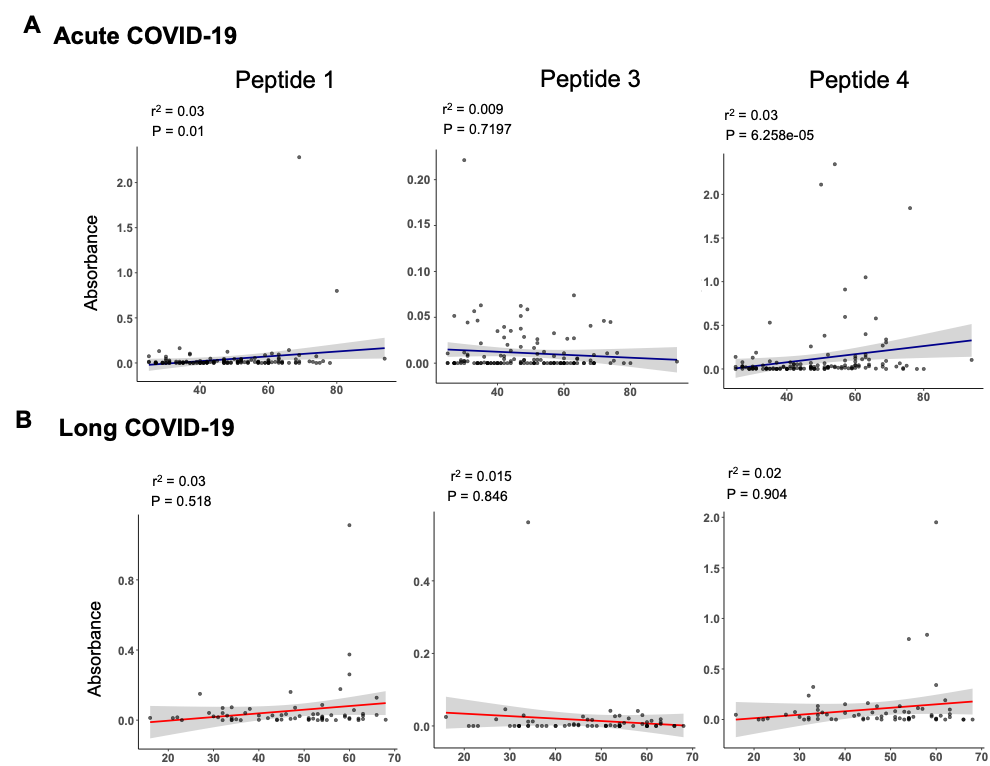


**Supplementary Figure S1.** Scatter plot showing the levels of serum reactivity towards each peptide in correlation with the age of patients. (A) Blue lines represent the linear regression of acute COVID-19 group and (B) Red line represent the linear regression of long COVID-19 group. Adjusted R square and P values from Kendall correlation shown on the top of each graph.

**
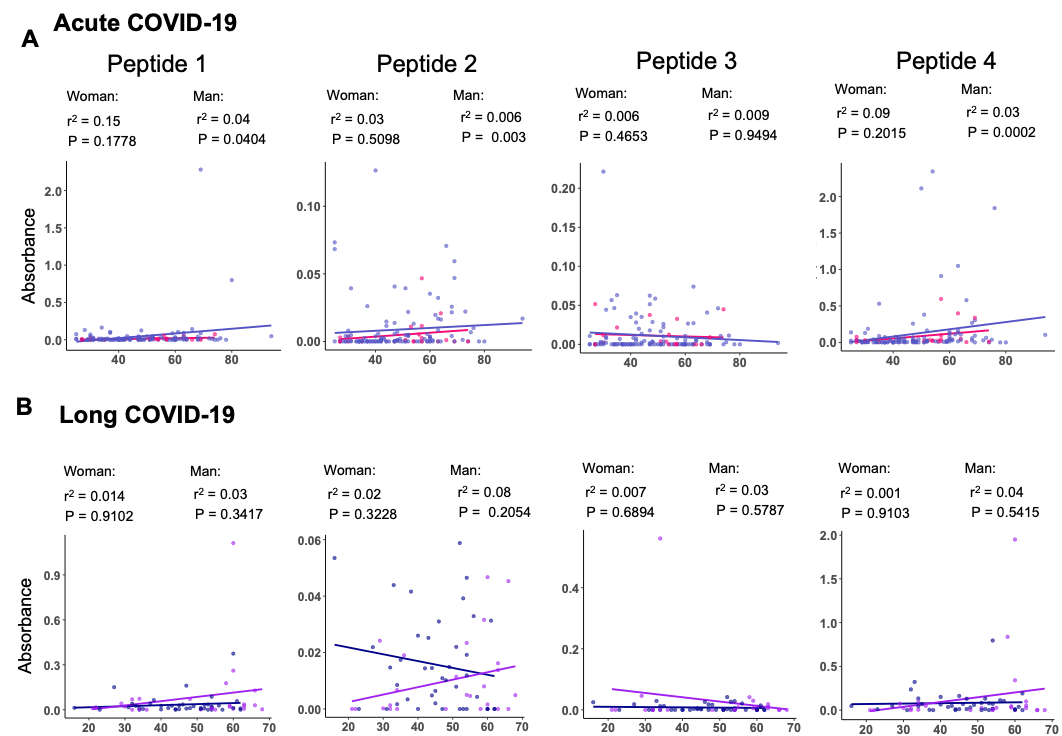
**

**Supplementary Figure S2.** Scatter plot showing the levels of serum reactivity in correlation with age and gender for each peptide. Dark blue and purple dots and linear regression lines represent man and woman with (A) acute COVID-19 and (B) long COVID-19. Adjusted R square and P values for the Kendall rank correlation coefficient shown at the top of each graph.

**References**

1. Zhang J, Yan R, Wu C, Wang H, Yang G, Zhong Y, Liu Y, Wan L, Tang A. *Spermatogenesis-associated 48* is essential for spermatogenesis in mice. *Andrologia* (2018) 50:e13027. doi: 10.1111/and.13027

2. Ris-Stalpers C, Trifiro MA, Kuiper GGJM, Jenster G, Romalo G, Sai T, Van Rooij HCJ, Kaufman M, Rosenfield RL, Liao S, et al. Substitution of Aspartic Acid-686 by Histidine or Asparagine in the Human Androgen Receptor Leads to a Functionally Inactive Protein with Altered Hormone-Binding Characteristics. *Molecular Endocrinology* (1991) 5:1562–1569. doi: 10.1210/mend-5-10-1562

3. Brüggenwirth HT, Boehmer ALM, Verleun-Mooijman MCT, Hoogenboezem T, Kleijer WJ, Otten BJ, Trapman J, Brinkmann AO. Molecular basis of androgen insensitivity. *The Journal of Steroid Biochemistry and Molecular Biology* (1996) 58:569–575. doi: 10.1016/0960-0760(96)00095-7

4. Marcelli M, Zoppi S, Grino PB, Griffin JE, Wilson JD, McPhaul MJ. A mutation in the DNA-binding domain of the androgen receptor gene causes complete testicular feminization in a patient with receptor-positive androgen resistance. *J Clin Invest* (1991) 87:1123–1126. doi: 10.1172/JCI115076

5. Yan W, Ma L, Burns KH, Matzuk MM. HILS1 is a spermatid-specific linker histone H1-like protein implicated in chromatin remodeling during mammalian spermiogenesis. *Proc Natl Acad Sci USA* (2003) 100:10546–10551. doi: 10.1073/pnas.1837812100

6. Capoano CA, Wettstein R, Kun A, Geisinger A. Spats 1 (Srsp1) is differentially expressed during testis development of the rat. *Gene Expression Patterns* (2010) 10:1–8. doi: 10.1016/j.gep.2009.11.006

7. Choi Y, Jeon S, Choi M, Lee M, Park M, Lee DR, Jun K-Y, Kwon Y, Lee O-H, Song S-H, et al. Mutations in SOHLH1 gene associate with nonobstructive Azoospermia. *Hum Mutat* (2010) 31:788–793. doi: 10.1002/humu.21264

8. Gu A, Ji G, Shi X, Long Y, Xia Y, Song L, Wang S, Wang X. Genetic variants in Piwi-interacting RNA pathway genes confer susceptibility to spermatogenic failure in a Chinese population. *Human Reproduction* (2010) 25:2955–2961. doi: 10.1093/humrep/deq274

9. Senoo M, Hoshino S, Mochida N, Matsumura Y, Habu S. Identification of a Novel Protein p59scr, Which Is Expressed at Specific Stages of Mouse Spermatogenesis. *Biochemical and Biophysical Research Communications* (2002) 292:992–998. doi: 10.1006/bbrc.2002.6769

10. Luk JM, Lee NPY, Shum CK, Lam BY, Siu AFM, Che C, Tam P, Cheung ANY, Yang ZM, Lin Y, et al. Acrosome‐specific gene *AEP1* : Identification, characterization and roles in spermatogenesis. *Journal Cellular Physiology* (2006) 209:755–766. doi: 10.1002/jcp.20746

11. Sujit KM, Singh V, Trivedi S, Singh K, Gupta G, Rajender S. Increased DNA methylation in the spermatogenesis‐associated (SPATA) genes correlates with infertility. *Andrology* (2020) 8:602–609. doi: 10.1111/andr.12742

12. Krotz SP, Ballow DJ, Choi Y, Rajkovic A. Expression and localization of the novel and highly conserved gametocyte-specific factor 1 during oogenesis and spermatogenesis. *Fertility and Sterility* (2009) 91:2020–2024. doi: 10.1016/j.fertnstert.2008.05.042

13. Huntriss J, Lu J, Hemmings K, Bayne R, Anderson R, Rutherford A, Balen A, Elder K, Picton HM. Isolation and expression of the human gametocyte-specific factor 1 gene (GTSF1) in fetal ovary, oocytes, and preimplantation embryos. *J Assist Reprod Genet* (2017) 34:23–31. doi: 10.1007/s10815-016-0795-0

14. Dirami T, Rode B, Jollivet M, Da Silva N, Escalier D, Gaitch N, Norez C, Tuffery P, Wolf J-P, Becq F, et al. Missense Mutations in SLC26A8, Encoding a Sperm-Specific Activator of CFTR, Are Associated with Human Asthenozoospermia. *The American Journal of Human Genetics* (2013) 92:760–766. doi: 10.1016/j.ajhg.2013.03.016

15. Røsok Ø, Pedeutour F, Ree AH, Aasheim H-C. Identification and Characterization of TESK2, a Novel Member of the LIMK/TESK Family of Protein Kinases, Predominantly Expressed in Testis. *Genomics* (1999) 61:44–54. doi: 10.1006/geno.1999.5922

16. Yatsenko AN, O’Neil DS, Roy A, Arias-Mendoza PA, Chen R, Murthy LJ, Lamb DJ, Matzuk MM. Association of mutations in the zona pellucida binding protein 1 (ZPBP1) gene with abnormal sperm head morphology in infertile men. *MHR: Basic science of reproductive medicine* (2012) 18:14–21. doi: 10.1093/molehr/gar057

17. Cheng LJ, Li JM, Chen J, Ge YH, Yu ZR, Han DS, Zhou ZM, Sha JH. NYD-SP16, a Novel Gene Associated with Spermatogenesis of Human Testis1. *Biology of Reproduction* (2003) 68:190–198. doi: 10.1095/biolreprod.102.004242

18. Jha KN, Coleman AR, Wong L, Salicioni AM, Howcroft E, Johnson GR. Heat Shock Protein 90 Functions to Stabilize and Activate the Testis-specific Serine/Threonine Kinases, a Family of Kinases Essential for Male Fertility. *Journal of Biological Chemistry* (2013) 288:16308–16320. doi: 10.1074/jbc.M112.400978

19. Sha J-H, Zhou Z-M, Li J-M, Lin M, Zhu H, Zhu H, Zhou Y-D, Wang L-L, Wang Y-Q, Zhou K-Y. Expression of a novel bHLH-Zip gene in human testis. *Asian J Androl* (2003) 5:83–88.

20. Liu C, Lv M, He X, Zhu Y, Amiri-Yekta A, Li W, Wu H, Kherraf Z-E, Liu W, Zhang J, et al. Homozygous mutations in *SPEF2* induce multiple morphological abnormalities of the sperm flagella and male infertility. *J Med Genet* (2020) 57:31–37. doi: 10.1136/jmedgenet-2019-106011

21. Chen A, Li J, Song L, Ji C, Böing M, Chen J, Brand-Saberi B. GGNBP2 is necessary for testis morphology and sperm development. *Sci Rep* (2017) 7:2998. doi: 10.1038/s41598-017-03193-y

22. Wojtas MN, Pandey RR, Mendel M, Homolka D, Sachidanandam R, Pillai RS. Regulation of m6A Transcripts by the 3ʹ→5ʹ RNA Helicase YTHDC2 Is Essential for a Successful Meiotic Program in the Mammalian Germline. *Molecular Cell* (2017) 68:374-387.e12. doi: 10.1016/j.molcel.2017.09.021

23. Matson CK, Murphy MW, Sarver AL, Griswold MD, Bardwell VJ, Zarkower D. DMRT1 prevents female reprogramming in the postnatal mammalian testis. *Nature* (2011) 476:101–104. doi: 10.1038/nature10239

24. Lima AC, Carvalho F, Gonçalves J, Fernandes S, Marques PI, Sousa M, Barros A, Seixas S, Amorim A, Conrad DF, et al. Rare double sex and mab-3-related transcription factor 1 regulatory variants in severe spermatogenic failure. *Andrology* (2015) 3:825–833. doi: 10.1111/andr.12063

25. Ferguson-Smith M. The Evolution of Sex Chromosomes and Sex Determination in Vertebrates and the Key Role of *DMRT1*. *Sex Dev* (2007) 1:2–11. doi: 10.1159/000096234

26. Koopman P. Sex determination: the power of DMRT1. *Trends in Genetics* (2009) 25:479–481. doi: 10.1016/j.tig.2009.09.009

27. Fedotkina T, Gurevich V, Muzalevskaya M, Normatov M, Churilov L. “Molecular mimicry between autoantigens of human endotheliocytes and Coronaviruses and promotion of atherogenesis.,” *In: 9th International Congress of Pathophysiology and 5th Congress of Physiological Sciences of Serbia with International Participation*
